# Retinitis Pigmentosa: Progress in Molecular Pathology and Biotherapeutical Strategies

**DOI:** 10.3390/ijms23094883

**Published:** 2022-04-28

**Authors:** Wanqin Liu, Shanshan Liu, Ping Li, Kai Yao

**Affiliations:** Institute of Visual Neuroscience and Stem Cell Engineering, College of Life Sciences and Health, Wuhan University of Science and Technology, Wuhan 430065, China; 18872419615@163.com (W.L.); lss1996633@outlook.com (S.L.); amy4115@163.com (P.L.)

**Keywords:** retinitis pigmentosa (RP), cell death, retinal remodeling, gene therapy, induced pluripotent stem cells, optogenetics

## Abstract

Retinitis pigmentosa (RP) is genetically heterogeneous retinopathy caused by photoreceptor cell death and retinal pigment epithelial atrophy that eventually results in blindness in bilateral eyes. Various photoreceptor cell death types and pathological phenotypic changes that have been disclosed in RP demand in-depth research of its pathogenic mechanism that may account for inter-patient heterogeneous responses to mainstream drug treatment. As the primary method for studying the genetic characteristics of RP, molecular biology has been widely used in disease diagnosis and clinical trials. Current technology iterations, such as gene therapy, stem cell therapy, and optogenetics, are advancing towards precise diagnosis and clinical applications. Specifically, technologies, such as effective delivery vectors, CRISPR/Cas9 technology, and iPSC-based cell transplantation, hasten the pace of personalized precision medicine in RP. The combination of conventional therapy and state-of-the-art medication is promising in revolutionizing RP treatment strategies. This article provides an overview of the latest research on the pathogenesis, diagnosis, and treatment of retinitis pigmentosa, aiming for a convenient reference of what has been achieved so far.

## 1. Introduction

Long evolution has allowed animals to obtain more than 80% of the information from the outside world through vision. In animals, as a separate structure in the brain, the eyeballs capture and process a wide range of visual information and transmit it along neurons to the brain for integration and editing. Thus, the health of the retina, which is responsible for the signaling and processing of this process, largely determines the state of vision. Deteriorating retinal function causes various visual system disorders, with retinitis pigmentosa (RP) being one of the most common and severe forms of this disease [1]. RP is an inherited retinal neurodegenerative disease characterized by progressive photoreceptor cell death and RPE atrophy, which initially manifests as nyctalopia, followed by continuous vision loss until blindness. The prevalence of RP ranges from 1/7000 to 1/3000 worldwide and is about 1/4000 in China; the age of onset of RP is early and yet to be precisely determined. In general, early-onset RP subtypes tend to progress rapidly. It usually starts around 10 years old; vision impairment is evident and refractory by the age of 40–50 [2]. It is generally accepted that early intervention slows disease progression. Therefore, early molecular diagnosis in combination with familial genetic information and specified phenotypic characteristics are indispensable for effective intervention. Nowadays, over a hundred pathogenic genes that act in various distinct biological pathways associated with RP have been identified since the advancement of molecular diagnosis [1]. Moreover, most of them are rod-specific, with only a tiny fraction of them in other retinal cells, such as the retinal pigment epithelium (RPE cells) [3].

The vast majority of genetically heterogeneous RP patients worldwide follow Mendelian laws of inheritance. The identified RPs are broadly divided into three categories according to the location and expression trait of the causative gene: autosomal-dominant RP (adRP, 15–25%) [4], autosome-recessive RP (arRP, 5–20%) [5,6], and X-linked RP (x-RP, 10–15%) [7]. In addition, 40–50% of the remainder have divergent phenotypic features, with bi-genetic RP and mitochondrial inherited RP being rare. In general, patients with X-linked RP exhibit more severe disease phenotypes than those with arRP, whereas patients with adRP [8] have the best prognoses with preserved central vision [9]. Also, the classification into syndromic RP and non-syndromic RP is based on the presence and absence of other physical defects; to be more specific, the latter manifests only ocular abnormalities. This review focuses on non-syndromic RP [10,11].

## 2. Introductory Eye Physiology

**Figure 1:**
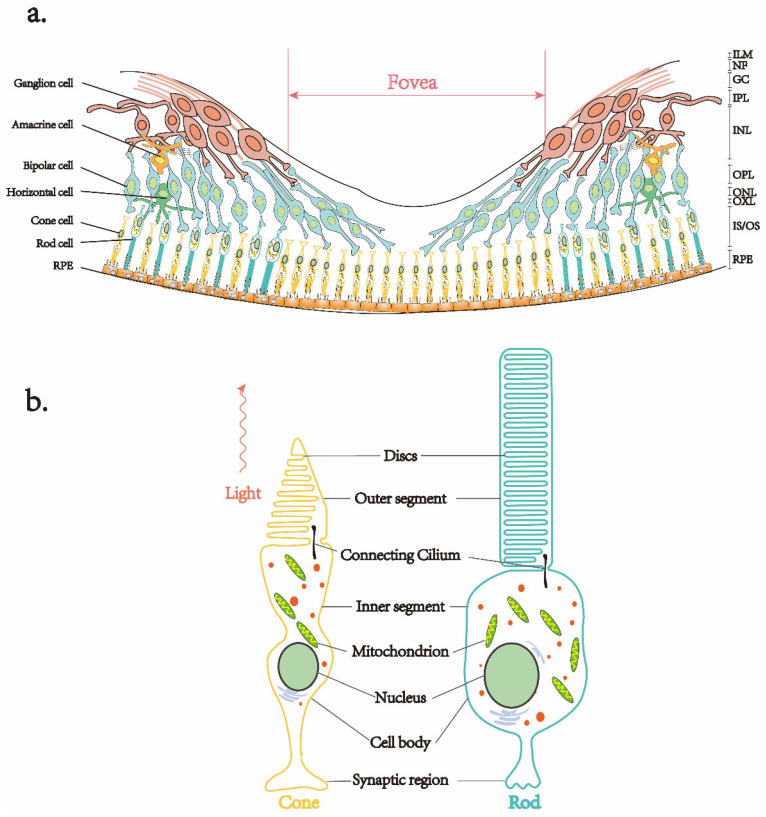
Retinal laminae and photoreceptor cell structure. (**a**) The retina consists of ten layers, of which photoreceptor cells (rod and cone) and retinal pigment epithelium (RPE) are the main target cells for the treatment of RP and other inherited retinal dystrophies. (**b**) The final morphological structure of photoreceptor cell development includes an inner segment, outer segment, and a synaptic terminal. Connecting Cilium transports components, such as proteins, from the inner segment to the outer segment to the sensory discs stacked in the outer segment in order to mediate the onset of light signal transduction.

Photoreceptor (PR) and RPE are the predominant cell types that suffer damage and undergo atrophy in retinal pigment degeneration [12]; both PR and RPE are closely interlinked and are crucial for phototransduction reactions (Figure 1). Early studies revealed that the final morphological structure of PR consists of three main parts: the inner segment, the outer segment, and the synaptic terminal [13]. The outer segment contains various proteins, such as retinoid and transducing, that are directly related to the phototransduction response. Rod photoreceptors are nutritionally complementary to cone photoreceptors, but they are interdependent [14]. The RPE, on the other hand, keeps renewing the PR outer segment on a daily and rhythmic basis, allowing the visual process to function stably [15,16,17]. The visual pigments located on the outer segment membrane are mainly composed of retinoids and chromophores, which are closely arranged. Each rod contains about 108 visual pigment molecules that mostly gather on the membrane disc separated from the outer membrane; in contrast, the cones gather at the inner fold attached to the cell membrane. The mammalian retina has only one type of rod—rhodopsin. Still, most mammals (including mice) have two types of cone opsin: S cone opsin (also known as blue-sensitive opsin) and M cone opsin (also known as green-sensitive opsin). Humans and primates have an additional L cone opsin (also known as red-sensitive opsin) that is sensitive to long wavelengths (red); together with S and M cone opsin, it produces trichromatic vision [13]. Human visual occurrence mainly relies on the cones that receive bright light stimulation and high-resolution color vision. Therefore, RP-related pathogenic gene mutations often simultaneously entangle color vision impairment. In addition, the migration of RPE cells to the neural retina observed through funduscopy eventually leads to osteoblast-like pigmentation in the advanced stage of RP [18].

## 3. Pathogenesis of RP

### 3.1. Cell Death in RP

**Figure 2:**
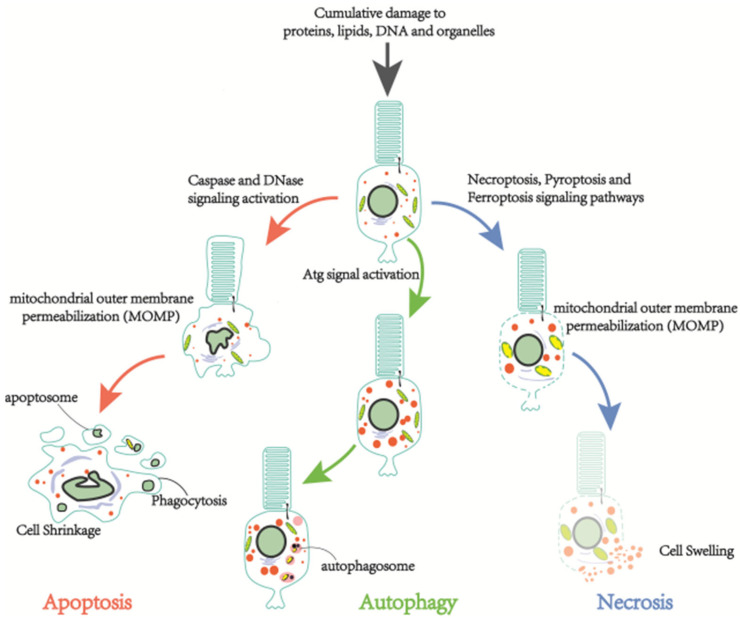
Three common types of cell death in RP. Damage signals from intracellular macromolecules cause necrosis, apoptosis, and autophagy, respectively.

Photoreceptor cell death is the ultimate cause of vision loss in retinitis pigmentosa. Together with autophagic and necrotic signals, dysregulated apoptosis is responsible for photoreceptor cell death [19] (Figure 2). Photoreceptor cells, along with other neuronal cells undergoing developmental apoptosis, are involved in the visual recycling system of the organism; they no longer divide or grow. However, they must survive for decades facing cumulative damage to proteins, lipids, deoxyribonucleic acid, and organelles. The above-mentioned post-mitotic nature of most neuronal cells allows the regulation of cell life beyond normal physiological death. It may also appear to induce the abnormal death of photoreceptor cells. Nowadays, over ten sorts of cell death have been classified by the Nomenclature Committee on Cell Death (NCCD). Cell death can be broadly divided into accidental cell death (ACD) and regulatory cell death (RCD). RP is primarily concerned with RCD; therefore, discrepant RP neuronal cell death types concerning signaling cascades, unique biochemical morphological features, and immunological consequences will be presented in this section.

#### 3.1.1. Apoptosis

**Figure 3:**
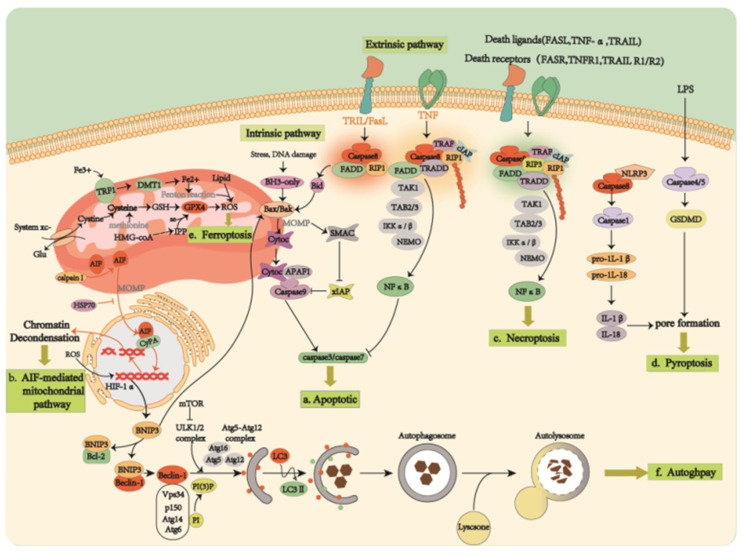
Part of the cell death mechanism. Schematic diagrams are represented as: (**a**) Activation of multiple protein complexes by caspase-8 induces caspase-dependent apoptosis; (**b**) AIF-mediated mitochondrial pathway induces caspase-independent apoptosis; (**c**) Necroptosis performed by RIPK1 and/or RIPK3; (**d**) Pyroptosis caused by the immune response activation of caspase family members; (**e**) Ferroptosis caused by the excessive oxidation of membrane lipids; (**f**) Atg family-mediated autophagy-dependent cell death.

Physiologically, RCD undergoes programmed cell death (PCD), also known as apoptosis. Photoreceptor cell apoptosis falls into two categories, caspase-dependent apoptosis and caspase-independent apoptosis [20]. The caspase family of proteins consist of pre-structural domains, p20, and p10 subunits. The apoptotic executioner caspases are responsible for the characteristic morphological changes caused by apoptosis, including membrane vesiculation, cell shrinkage, the formation of “apoptotic bodies”, and chromosomal deoxyribonucleic acid breakage [21]. According to their function, the caspase family are subdivided into initiator caspases (caspases-1, -2, -4, -5, -8, -9, -10, -11, and -12) and effector caspases (caspases-3, -6, and -7). Briefly, the inhibitor caspase first activates a multiprotein complex, followed by the activation of a downstream effector caspase that cleaves a broad spectrum of protein substrates, thereby inducing apoptosis and other biological functions. Four complexes have been found to be activated by caspases to induce apoptosis [19]. The first complex is the death-inducing signaling complex; it is involved in both endogenous and exogenous pathways [22] (Figure 3a). The exogenous apoptotic pathway is triggered by the attachment of tumor necrosis factor (TNF) to the cell surface TNF family death receptors that lead to the recruitment of the Fas-associated death structural domain protein (FADD). FADD, in turn, binds the pre-cysteine-8 molecule, allowing for an auto-protein hydrolysis process and the activation of cysteine-8 [23]. The activated caspase-8 cleavage activates either downstream caspases or only the BH3 receptor-containing pro-apoptotic Bcl-2 family protein, Bid, which subsequently activates Bax to mediate the mitochondrial outer membrane permeabilization (MOMP), with the resultant release of mitochondrial proteins and cytochrome C [23]. Some studies have shown that caspase-10 appears to exhibit pro-apoptotic properties, as well as promote NF-kB activation and cell survival via autophagic and apoptotic pathways [21,24]. In contrast, the internal (mitochondrial) pathway is triggered intracellularly and is mediated by ATP. To be more specific, the released cytochrome C bound to APAF-1 activates caspase-9 to form apoptotic vesicles (a second complex) to cleave and activate downstream caspases that degrade cellular proteins [25,26]. The other two complexes are PIDD vesicles and inflammatory vesicles. While PIDD vesicles activate up-stream protein caspase-2 in the endogenous mitochondrial pathway [27], inflammatory vesicles activate caspase-1 to promote the cleavage of IL-1b and IL-18 into their mature pro-inflammatory forms.

The apoptosis-inducing factor (AIF)-mediated mitochondrial pathway (Figure 3b), on the other hand, is a caspase non-dependent apoptosis [20]. AIF is a flavin protein located in the mitochondrial intermembrane; it is involved in energy and redox metabolism [28]. It has been found that cleaved AIF is transferred to the cytoplasm and nucleus under stress conditions to disassemble chromatin. The cleavage of AIF is regulated by a variety of molecules and signals, such as the key enzyme calpainⅠ (m-calpain) that mediates AIF processing [29,30,31]. The transfer of AIF is a two-step process: first, AIF is released to the cytoplasm via MOMP and cleavage in intermembrane space (IMS); second, AIF is translocated to the nucleus through the interaction between AIF and procyclin A, which was observed in dying optic rod cells in mouse and rat models of RP [32]. These findings suggest that AIF translocation and its regulatory pathways mediate apoptosis in photoreceptor cells.

#### 3.1.2. Necrosis

The sequential discovery of multiple modifiable mechanisms involved in necrosis during the last decade holds promise for targeted therapies for RP. Neuronal cell necrosis includes necroptosis, pyroptosis, and ferroptosis [33]. The well-understood necroptosis is programmed cell death, similar to cell necrosis (Figure 3c). It is executed by RIPK1 and/or RIPK3 when cystatin proteases are inhibited. RIP1 is a multifunctional bridging protein located downstream of the death receptor; it mediates the NF-k8 activation of pro-survival properties, cystatin-dependent activation, and RIP kinase-dependent necrosis. Death signaling also activates RIPK3 kinase, which phosphorylates MLKL, the specific executive protein of cellular necrosis. Then, the phosphorylated MLKL (p-MLKL) undergoes oligomerization and is translocated to the cellular membrane, which leads to its disruption and resultant cell death and leakage of intracellular material. Using transmission electron microscopy, studies of RP patients with extensive optic rod degeneration revealed abnormal morphology in the remaining retinal cells: swollen cytoplasm, ruptured plasma membrane, and autophagic vacuoles. These findings suggest that non-apoptotic mechanisms may be involved in the secondary death of retinal cells [34]. In a mouse model of rod-specific gene mutation, RIP kinase-mediated necrosis was responsible for retinal cell death [35]. The above findings suggest that targeting both necrotic and endogenous anti-apoptotic pathways may be a potential therapeutic approach for retinal degenerative diseases. Neuronal cell pyroptosis might result from pathogen-associated molecular patterns (PAMPs), damage-associated molecular patterns (DAMPs), and other inflammatory signals that selectively activate caspase family members. The activated caspase proteins cleave the GSDMD into GSDMD-N, which is then partially translocated onto the inner leaflet of the plasma membrane and binds to phospholipids that lead to pore formation and ultimate cell lysis. It usually incurs an inflammatory response within the retina [33] (Figure 3d). Another example is oxidative stress-induced ROS signaling activation that induces the assembly of NLRP3 inflammatory vesicles [36]. Ferroptosis is regulated cell death caused by lipid peroxidation; it differs from other types of cell death at the genetic, biochemical, and morphological levels [37,38]. Glutathione peroxidase 4 (GPX4) plays a critical role in the prevention of excessive lipid peroxidation in various cells, including neurons. The apoptotic genes AIF and GPX4 levels were significantly elevated in oxidant-induced retinal degeneration. The protective effect of GPX4 on photoreceptor cells has been well-observed, but the exact mechanism warrants further investigation [39]. Some studies have shown that GPX4 not only protects photoreceptor cells [40], but is also a critical antioxidant enzyme for the maturation and survival of photoreceptor cells [41]. GSH depletion leads to oxidative stress and lipid peroxidation; whether iron cell death is related to GSH depletion in RPE is currently unknown [42] (Figure 3e). To recap, each of these necrosis types in RP is yet to be well understood, which might underlie the mechanism for potential therapeutic target exploration. 

#### 3.1.3. Autophagy-Dependent Cell Death

Autophagy is a major intracellular catabolic system; it is involved in the physiologically dynamic recycling of cells and pathological conditions such as neuronal degeneration [43]. Autophagy-dependent cell death is an RCD characterized by autophagic vacuolization, a pathological feature involving the formation and recycling of autophagosomes (Figure 3f). Since the identification of *Atg* genes that are essential for autophagy induction in yeast [44], significant progress has been made in the molecular mechanisms of autophagy [45]. In the induction of autophagy, Atg6, together with Atg14, vacuolar protein-sorting Vps34, and p150/Vps15, forms the initial morphology of the isolation membrane after class-III PI3-kinase activation; the extension of the isolation membrane requires two ubiquitin-like binding systems: the Atg5–Atg12 binding system and the LC3 binding system. First, the Atg12–Atg5 adducts, and then the mammalian homolog LC3 (a microtubule-associated protein) is recruited into the isolation membrane, which isolates cytoplasmic material [46]. LC3 within the isolation membrane is then cleaved by the cysteine protease Atg4 to become LC3-I [47], which is later adducted with the membrane-bound phosphatidylethanolamine (PE) to form LC3-II, a marker of autophagosome formation [48]. After that, Atg4 uncouples LC3-II, which is, therefore, released from the membrane to be recycled or degraded by lysosomal enzymes in the autolysosome. Studies have disclosed LC3-II level upregulation following photodamage and H_2_O_2_ treatment to 661W photoreceptor-like cells in the mouse retina; treatment with 3-MA and knockdown of Atg5 and Beclin 1 partially blocks H_2_O_2_-induced 661W cell death [49], suggesting that autophagy may contribute to oxidative stress-induced photoreceptor cell death. In a mouse model of RP, it was also confirmed that rod photoreceptor cells die mainly of apoptosis; the ensuing cone cell death displays necrotic features and the accumulation of autophagic vacuoles [35].

### 3.2. Phenotypic Switch in RP

**Figure 4:**
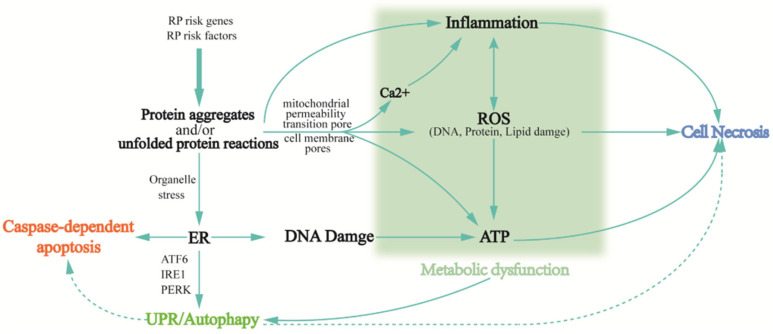
Biochemical reactions, such as protein aggregation, oxidative stress, the immune response, and metabolic dysfunction that occur during retinal degeneration cause retinal cell death. When gene mutations trigger macromolecular aggregation, one leads to endoplasmic reticulum stress, activating the unfolded protein response (UPR), but when UPR activation is not sufficient to relieve stress, cell death is induced by activating pro-apoptotic pathways (e.g., Caspases activation, Ca^2+^ release, and mitochondrial signaling); second, the oxidative system and antioxidant system imbalance and cyclically aggravate retinal oxidative stress. Third, it triggers the mechanism’s immune defense, when the active markers are immune cells and immune factors. The result of these unsustainable reactions ultimately points to the death of photoreceptor cells in various ways. Another pathway of cell death is autophagy, while excessive autophagy triggers apoptosis and necrosis. In addition, the accumulation of these reactions to a certain extent causes metabolic dysfunction, which is also closely associated with autophagy.

Retinal degeneration is associated with fundamental phenotypic changes, such as photoreceptor cell death, oxidative stress, immune responses, and metabolic dysfunction. When pathogenic RP genes begin to be translated sustainedly, a series of modifying responses are elicited in response to the mutant protein, known as retinal remodeling. During this process, the inner retina undergoes three dynamic phases: first, the initial stress-induced degeneration of photoreceptor cells leads to neural reprogramming (neural reprogramming) and glial responses, such as inappropriate localization and expression of ON-BC and OFF-BC receptors, and topology disruption of normal neuronal as well as altered metabolic properties of Muller cells [18,50,51]. The microscopically drastic changes in the retinal layer are followed by the continued loss of the remaining photoreceptor cells (especially cone cells), dysregulated immune responses, and increased oxidative stress. Therefore, the final stage is characterized by the complete loss of photoreceptor cells and chronic inflammation due to the abnormal metabolic characteristics of immune cells. Generally, the stress response triggers either a protective immune defense, where coordinated immune cells work to resolve inflammation, or the imbalance between oxidative and antioxidant systems, aggravating retinal oxidative stress. A response of the latter kind ultimately points to the death of photoreceptor cells in various ways. Another cell death pathway is autophagy, which is normally the mechanism by which the body removes harmful reactants; however, excessive autophagy induces abnormal apoptosis and necrosis, as well as metabolic dysfunction. Moreover, degenerated retina composition analysis has revealed that massive intracellular protein aggregates are featured in photoreceptor cell death [52]. These protein aggregates are closely linked to cellular biochemical responses, such as innate immunity, oxidative stress, and autophagy, that arise during retinal remodeling (Figure 4).

#### 3.2.1. Protein Aggregation and/or Unfolded Protein Reactions

Protein aggregates are the leading cause of neuronal dysfunction and even death in neurodegenerative diseases. Tau and amyloid, two well-studied aggregates, are responsible for Alzheimer’s disease and alpha-synuclein in Parkinson’s disease. In RP, several protein aggregates have also been unveiled chronologically. Most of them are specific proteins involved in the visual cycle within photoreceptor cells. For example, misfolded proteorhodopsins result from single base substitution in P23H and lead to the endoplasmic reticulum (ER) stress and activation of the unfolded protein response (UPR) with ensuing protein aggregation [53]. Insufficient UPR activation that is unable to relieve stress in ER, in another way, activates pro-apoptotic pathways (e.g., Caspases activation, Ca2+ release, and mitochondrial signaling) [54,55]. Protein aggregates also trigger degenerative signals in the neurons. Moreover, as these protein aggregates are highly ubiquitinated, they also lead to the impairment of the ubiquitin protease system that deteriorates the cellular environment [56]. In addition, mutant forms of α-synuclein (α-syn) are prone to pathologically aggregate [57] to permeabilize membrane bilayers, leading to calcium overload, oxidative stress, mitochondrial permeabilization [58,59], and final apoptosis or necrosis. Utilizing the phosphorylation of serine at 129 (Pα-syn), the specific marker of α-syn lesions, to evaluate the distribution of α-syn across aging timepoints in the rhodopsin transgenic (Tg) P347L rabbit that was established for retinal remodeling observation, it was disclosed that both the distribution and expression levels of α-syn and Pα-syn in the Tg retina varied significantly as it progresses, which can be explained by aggravated retina remodeling [52].

#### 3.2.2. Inflammatory Response

In retinopathy, the innate immune response is a frontline driver in the pathogenesis of RP. The innate immune system includes physical and chemical barriers (humoral and cellular immunity) that maintain the balance of the internal environment and prevent microbial invasion. It also activates the adaptive immune response that aids the elimination or amplification of immune responses when appropriate [60]. After breaching the physical barrier, stress-related factors are first encountered by innate immune cells that actively induce and regulate inflammation to prevent profound tissue damage. Extensive studies on the differences of CD antigens between microglia and macrophages in RP animal models [61,62] have revealed that, although the main form of the mutation-induced pathway of rod death is apoptosis, which manifests as cytoplasmic condensation and nuclear lysis, there is also significant upregulation of pro-inflammatory cytokine and chemokine (IL-1 α, IL-1β, IL-2, IL-4, IL-6, IL-8, IL-10, IFN-γ, GRO-α, I-309, IP-10, MCP-1, MCP-2, and TARC), as well as significantly increased microglia/macrophages [35]. Therefore, more attention is beginning to be devoted to the inflammatory response. Gradually, increased microglial activity is an early marker for various retinal degenerative diseases; it responds to retinal stress and cell death that initiate chronic inflammatory responses [63]. Neuroinflammation development is closely associated with the imbalance between oxidative DNA damage and its defense system that results in photoreceptor cell degeneration, accompanied by increased microglial activity [64]. However, the homeostatic balance in the phagocytosis of apoptotic photoreceptors by microglia is achieved by the C3-CR3 complement activation system [65]. Massive pro-inflammatory cytokines that have been observed to accumulate in the vitreous cavity of RP patients serve as potential inflammatory biomarkers. It has been disclosed that the monocyte chemotactic protein-1 (MCP-1) levels are significantly upregulated in human RP vitreous. MCP-1 promotes photoreceptor cell apoptosis via the microglia/macrophage activation pathway once retinal detachment occurs. Associated microglia phenotypes in apoptotic rods have also been identified. These phenomena suggest that MCP-1 is a candidate biomarker for monitoring disease progression [66]. In addition, the DAMPs (damage-associated molecular patterns, including ATP, HMGB1, S100 protein, HSP, DNA, RNA, etc.) produced by tissue injury or cellular stress may also inhibit or promote chronic inflammation. For example, extracellular adenosine triphosphate (ATP) is a crucial chemotactic signal that recruits innate immune cells to the site of retinal injury [60]. In a recent study, it was discovered that the RPE lipofuscin fluorophores N-retinylidene-N-retinylethanolamine (A2E) under long blue light treatment increased the reactive oxygen ROS levels, causing the upregulation of the expression of 26 pro-inflammatory cytokines and finally leading to retinal RPE degeneration. This reveals a connection between retinal degeneration and oxidative stress-induced immune cascade responses [67].

#### 3.2.3. Oxidative Stress

Oxidative stress (OS) is a critical feature of many pathological neurodegenerative lesions. As part of the normal functioning of the nervous system in the brain, the integrity of the Blood–Brain Barrier (BBB) is of great importance. Direct damage to the BBB from oxidative stress can affect the composition of the neurovascular unit (NVU), further exacerbating blood–brain barrier damage and dysfunction, ultimately leading to neuronal dysfunction, neuroinflammation, and neurodegenerative lesions [68,69,70]. In diseased retinas, the imbalance between oxidative and antioxidant systems leads to massive reactive oxygen species (ROS) production, including superoxide (^1^O_2_), hydrogen peroxide (H_2_O_2_), and hydroxyl radicals (-OH) [71]. In addition to direct cellular damage, multiple OS and anti-OS pathways activated by ROS also indirectly aggravate or reduce the degree of retinal damage [72]. The primary source of ROS in vivo is the mitochondria. Oxidative phosphorylation in the mitochondria generates small-molecule compounds that act in cell death pathways, including apoptosis, autophagy, and necrosis, as well as ROS that induce oxidative damage and even activate the autophagic pathway [73]. Another important source of ROS is NADPHylated enzymes, which might be enormous in quantity in phagocytes compared with that in other tissue cells. They are involved in the downstream signaling activation of various membrane receptors [74]. Considerable evidence from animal models and RP patients has revealed that excessive oxidative stress of macromolecules (lipids, proteins, and nucleic acids) increases retinal damage. Nowadays, the oxidative stress levels can be determined through detecting ROS signals simply by probe detection, such as dihydroethidium (DHE) and fibrinase in rd1 mice. To be more specific, macromolecules can be detected separately according to their oxidized residue specificity to the probe [75,76]. Retinal degeneration was significantly improved after treatment with antioxidants in several different mouse models [77,78,79]. The same could be achieved by modifying the relevant genes, the adeno-associated virus (AAV) vector-mediated delivery of nuclear factor erythroid-derived 2-like 2 (NRF2); this is a transcription factor that enhances detoxification and antioxidant genes in response to oxidative stimulation and is effective for retinal cone survival [80]. Analysis of atrial fluid, vitreous, and peripheral blood samples for macromolecular marker content has indicated abnormal oxidative phosphorylation levels within the retina of RP patients. The three studies mentioned above suggest that oxidative stress is crucial in retinal degeneration in retinopathy [81,82].

#### 3.2.4. Autophagy

In neurodegenerative diseases, autophagy-induced cell death caused by inflammation [83], mitochondrial stress [84,85], and protein misfolding/aggregation [73] are more predominant [86]. Low-level autophagy promotes the self-renewal of damaged neuronal cells in vivo and maintains neurological homeostasis, whereas excessive autophagy brings massive oxidative stress and protein aggregation in many neurodegenerative diseases, which further enhance autophagy and ultimately lead to the apoptosis and necrosis of neuronal cells [87]. Autophagy is vital to homeostasis maintenance in the face of a robust immune response by exquisitely inducing or inhibiting multiple immune mediators [88]. Inhibiting autophagy alleviates retinal degeneration caused by improper protein folding in deteriorated retinas; P23H (a mutant of RHO) mutant mice typically exhibit elevated levels of autophagic flux, compared to that of pharmacologically reduced or Atg5 (the rod-specific autophagy-activating gene)-silenced autophagic flux. The photoreceptor structure and function are better preserved in the latter two [73]. Thus, regulated autophagy is critical in maintaining cell survival. When cells lack nutrients, autophagy is moderately activated to serve as a nutrient source to sustain essential metabolic activities; simultaneously, it can trigger additional apoptosis [89]. The regulatory role of autophagy in RPE growth and metabolism has been extensively studied through autophagy-associated regulatory factor-deficient mouse models; they indicated that the daily autophagic requirement of RPE is regulated by precise genetic regulation for the digestive cycle of intracellular photoreceptor outer segment (POS) components under light and stress conditions [90].

#### 3.2.5. Metabolic Dysfunction

As one of the most energy-demanding tissues, the retina is extremely vulnerable to dysfunction in its metabolic energy system. RP-related excess apoptosis, necrosis, and autophagy cause disruption to both extracellular milieu homeostasis and intracellular metabolism, and result in massive neurotoxicity accumulation (imbalance of glutamate regulation). Mitochondria are essential for cellular metabolism—mitochondrial oxidative phosphorylation generates energy in processes such as calcium uptake and cellular metabolism; it also produces small molecule compounds that regulate the cell death pathways (including AIF, ROS production, MOMP, etc.) [73]. Pathogenic mutations in rods lead to retinal degeneration, followed by cone apoptosis and necrosis caused by metabolic dysregulation. The mTORC1 activation in the cones enhances glucose uptake, retention, and utilization, thereby increasing the NADPH levels, a crucial metabolite capable of mitigating retinal cell death [91]. The associated mTOR signaling pathway is a major negative regulator of autophagy. Pan and colleagues found that UXT (ubiquitously expressed prefoldin like chaperone)-deficient mice exhibited retinal degeneration and pigmentation. UXT inhibits apoptotic photoreceptor cell death by up-regulating the mTOR pathway; UXT knock-out promotes autophagic flux and apoptosis in photoreceptor cells [92]. Recently, mitochondrial and metabolic dysfunction has been found to be driven by the unifying mechanism—epithelial–mesenchymal transition (EMT) [93]. EMT is also featured in fundus retinopathy, especially retinal fibrotic diseases, such as subretinal fibrosis, in age-related macular degeneration (AMD) [94], wherein PGC-1α inhibits mitochondrial biogenesis and metabolic functioning in human retinal pigment epithelial cells [37,95].

### 3.3. Aberrant Biochemical Reaction in RP

Apart from some of the biochemical reactions that occur in cells, the life activities involved in RP will also be covered in the following section. RP-related genes are involved in five major biological activities, including phototransduction cascade reactions, RNA splicing, retinal transcription factor regulation, retinal cytoarchitectonic and functional regulation, and retinal metabolism [1]. Under physiological conditions, the signaling pathways featured in these activities interact to form a complex network that allows the accurate regulation of the signaling transduction cascade upon stimulation from the outside world.

#### 3.3.1. Phototransduction Cascade Reaction

The retina is responsible for the early stage of the light-induced neuronal signaling processes that generate the perception of objects, backgrounds, motion, shadows, and colors. Both opsin and rhodopsin are G protein-coupled receptors (GPCR) and are members of the cellular signaling protein family. With dim light, adenosine-gated cation channels open and continuously release glutamate to OFF-bipolar cells, depolarizing the optic rods; with bright light, the rhodopsin acts on G proteins to dephosphorylate GTP into GDP, which, together with the activation of G proteins whereby α subunit disassociates, activates phosphodiesterase (PDE) that later hydrolyzes cGMP. Without light stimulation, the cGMP concentration gradually decreases, accompanied by channels closing and rods hyperpolarizing. The RHO gene encodes rhodopsin; RHO mutation is thus believed to be pathogenic, which is also one of the leading causes of RP [96,97]. The AAV-mediated delivery of a highly efficient shRNA combined with a siRNA-resistant human RHO replacement cDNA has recently been proven to successfully delay the onset of photoreceptor cell degeneration in the RHO-T4R canine model, the only currently available animal model for RHO-adRP [98]. Unfolded or incorrectly folded rhodopsin retained in the endoplasmic reticulum generates organelle stress and activates the UPR; the insufficient activation of the UPR induces cell death through the activation of pro-apoptotic pathways, such as Caspase activation, Ca2+ release, and mitochondrial signaling [54,55]. It has been reported that the overexpression of heavy-chain binding protein (BiP) promotes the translocation of P23H rhodopsin to the cell membrane and reprograms the UPR in Rho P23H mutant rats, which was proven to inhibit the apoptosis of photoreceptor cells [53]. Since the discovery of channelrhodopsin with spectral properties, its unique photosensitive properties have been developed as an essential tool in modern biology, especially in the study of optogenetic techniques [99].

#### 3.3.2. RNA Splicing

RNA splicing is indispensable in human mRNA maturation. The spliceosome comprises functional proteins, such as the RNA-protein complex containing the precursor mRNA, the five small nuclear ribonucleoprotein particles (snRNP)-U1, U2, U4/U6, and U5, and many non-snRNP protein factors. Mutations in RNA splicing-related genes in retinopathy are broadly classified as the second-largest category of adRP, the first being associated with retinal mutations [100]. The splicing factor PRPF31 involves the interaction between U4/U6 di-snRNP and U5 snRNP [101,102]. PRPF31 knockdown by siRNA results in retina-specific mRNA gene down-expression. There are other splicing factor-related genes, such as RNA processing genes (PRPF8, PRPF3, PRPF4, and PRPF19), phototransduction genes (RHO, GNAT1/2, and RP1), photoreceptor cell structure genes (ROM1, FSCN2, and SEMA4), and transcription factors (CRX) [102,103]. Some non-snRNP splicing factors, such as DHX38 and CWC27, are associated with arRP [104,105]. The knockdown of CWC27, an unidentified splicing factor, or CWC22, a binding protein of CWC27, in immortalized retinal pigment epithelial cells witnessed upregulation in inflammation-related gene expression and downregulation in mitochondrial enzyme-related gene expression that is involved in oxidative phosphorylation, which subsequently induces immune responses and oxidative stress [106].

#### 3.3.3. Transcription Factor Regulation

During photoreceptor differentiation, transcription factors are known to regulate crucial processes of photoreceptor differentiation. Herein, we list several classical ones. The paired-type homodomain transcription factor OTX2, which regulates photoreceptor cell production, is expressed in the final stage of mitosis and early phase of photoreceptor precursor cells of retinal progenitor cells. The knockdown of OTX2 in immature retinal precursor cells resulted in almost complete loss of rods and cones [13]. Neural retina leucine zipper protein (NRL) determines whether or not rods can be generated. Studies have shown that interplay between NRL and cone-rod homeobox protein (CRX), together with other transcription factors, induces rod-specific gene expression [13,107] that promotes the development of undifferentiated rods [108]. Recent studies have indicated that NR2E3 inhibits the differentiation of retinal progenitor cells into cones during mitosis, while the NRL/NR2E3 pathway is associated with the differentiation and maintenance of rods throughout the cell life cycle. Therefore, the NRL pathway serves as a therapeutic target for the treatment of RP [109]. Transcription factor regulation also embodies the genetic heterogeneity of the disease. Microphthalmia-associated transcription factor (MITF) is essential in RPE development and function. Studies have found that retinal oxidative damage in *Mitf+/−* mice can be attenuated by the specific overexpression of NRF2, a significant regulator of antioxidant signaling, in retinal pigment epithelial cells. As MITF directly regulates NRF2 transcription and its translocation into the nucleus, it can be assumed that MITF might be a potential therapeutic target [110].

#### 3.3.4. Cellular Structure and Function Regulation

Photoreceptor cells in vertebrates have featured cellular structures, including optins and modified cilia composed of basal bodies, connecting Cilium, and outer segments (OSs); signal-transduction complexes that mediate phototransduction are distributed on photoreceptor discs stacked with the outer segments of cilia [111]. OSs lack biosynthetic functions, so they are synthesized and partially pre-assembled in photoreceptor intracellular segments (ISs), and are then transported to the outer segments by connecting cilia, which is facilitated by the intraflagellar transporter (IFT). IFT, together with non-syndromic retinopathy-associated pathogenic proteins (https://sph.uth.edu/retnet/home.htm, accessed on 20 April 2021), also assembles and maintains cilia [112,113], ensuring the integrity of cellular structures. The PRPH2 gene that encodes peripheral protein 2 is a retina-specific transmembrane glycoprotein hampering the development of the outer segments of the rods and cones (ROS and COS, respectively) [114]. A study has detected three missense mutation types in PRPH2 that affect the C-terminal structural domain of the PRPH2 protein, causing photoreceptor outer segment instability in clinical cases of leukoplakia retinitis pigmentosa (RPA) and confirming the potential disease-related mutations of PRPH2 by haplotype statistical analysis [115]. Rom1 is also a protein essential for regulating photoreceptor disc morphogenesis and maintaining mammalian photoreceptor cell activity. It is localized at the edge of the photoreceptor disc that fine-tunes its size and structure, and is essential for material renewal and structure maintenance within the Oss [116,117,118]. *Rom1−/−* mouse rods have a highly irregular OS morphology, which subsequently undergoes progressive apoptosis [116]. Moreover, considerable evidence suggests that the formation of the *Prph2* and *Rom1* complex dominates the development of the disc edge region of photoreceptor Oss [119], which undoubtedly increases the heterogeneity of retinal diseases [120]. CRB1, also known as RP12, is a transmembrane protein; it regulates the apical-basal polarity of retinal photoreceptor cells and controls the adhesion and relative position between cells in the retina [121,122]. CRB1 mutation-associated CRB complex alteration interferes with the retinal histogenesis process, resulting in mild to severe impairment of retinal vision in mice [123]. The photoreceptor interstitium fills the space between the photoreceptor cells and the RPE; it also aids the structural occurrence and metabolism of the retina, as well as cellular communication, photoreceptor alignment, and the adhesion of the retina to the RPE [124,125]. 

#### 3.3.5. Retinal Metabolism

Normal retina metabolism underlies vision formation; metabolic disorders lead to retinopathy. Various forms of disorders have been discovered, including glycolytic metabolism, polyol metabolism, amino acid metabolism (e.g., glycine, serine, and threonine metabolism; taurine and hypotaurine metabolism, etc.), and lipid metabolism (e.g., phospholipid metabolism, sphingolipid metabolism, glyceride metabolism, and fatty acid metabolism). Biological activities, such as glycolytic metabolism to meet the nutritional supply of the cones [126], the production of retinol in the visual cycle [127], and the regulation of lipid signaling pathways in the RPE [128], inevitably rely on retinal metabolism. Many important regulatory molecules, such as enzymes, hormones, and ligands, are involved in these reactions. Lecithin–retinol acyltransferase (LRAT), the main acyltransferase involved in the visual cycle, catalyzes the formation of retinyl esters by transferring acyl groups from the sn-1 position of phosphatidylcholine (PC) to vitamin A. The binding form of retinol-binding protein 4 (RBP4) and all-trans-retinol, holo-RBP4, is the major transport carrier of vitamin A in the blood, with LRAT enhancing the transfer efficiency of STRA6-dependent all-trans-retinol from holo-RBP4 to target cells [129]. Furthermore, the complete inactivation of the LRAT enzyme in human P173L-LRAT mutants leads to reduced night vision in infancy and triggers the loss of the visual field by the age of 60 years [130].

## 4. Clinical Manifestations and Diagnosis of RP

Physiological features of early retinal degeneration are manifested by the onset of photoreceptor cell stress and the beginning of outer segment shortening [52]. The initial clinical manifestation is the decrease in night vision, which is normal or near-normal on conventional fundus examination, but some early blanket-layer-like retinal degeneration can be noticed on mid-peripheral fundus photography. As the disease progresses, the visual field is gradually lost in a concentric pattern, with the progressive loss of the outer segment of the central concave retinal cone cells and extensive loss of photoreceptors in the peripheral region of the retina. Indeed, it is possible to target the RP-related gene or replace the damaged cells, depending on the disease course of RP. Over the years, the exploration of therapeutic approaches for hereditary retinitis pigmentosa has expanded considerably. Efforts have been made to translationally apply these approaches that may slow down photoreceptor degeneration or restore vision in the clinic. Simultaneously, the real-time status of the disease needs to be evaluated before and during subsequent treatment.

Based on multiple sources of evidence, we obtained an evaluation form that provides a good overview of the current clinical evaluation process and RP diagnosis (Table 1). At the initial visit, a complete ocular history and a genealogy documenting the family history of eye disease should be obtained first and updated at subsequent visits. The ensuing clinical evaluation includes a clinical ophthalmologic examination, funduscopy (OCT), visual field testing (VF), and electroretinography (ERG). Finally, molecular genetic testing is required, as the genotyping of patients and families is a prerequisite for diagnosis confirmation, better consultation, and individualized treatment. To achieve precise disease staging and inform of a possible diagnosis, a correct diagnosis with additional and improved diagnostic tools is indispensable. In conclusion, a good clinical diagnosis will be beneficial in guiding the treatment of patients with RP. Table 1 illustrates the RP clinical evaluation and diagnostic status.

## 5. Therapeutic Approaches to RP

Although RP is genetically heterogeneous and pleiotropic, with numerous pathogenic mutations leading to an extremely complex clinical presentation, the common result is photoreceptor apoptosis and retinal damage following RPE degeneration. As the disease progresses, the most effective treatment for the different stages depends on the number of remaining photoreceptor cells [99]. Treatment modalities that are frequently studied clinically today are discussed in the following.

### 5.1. Neuroprotective Agent

**Figure 5:**
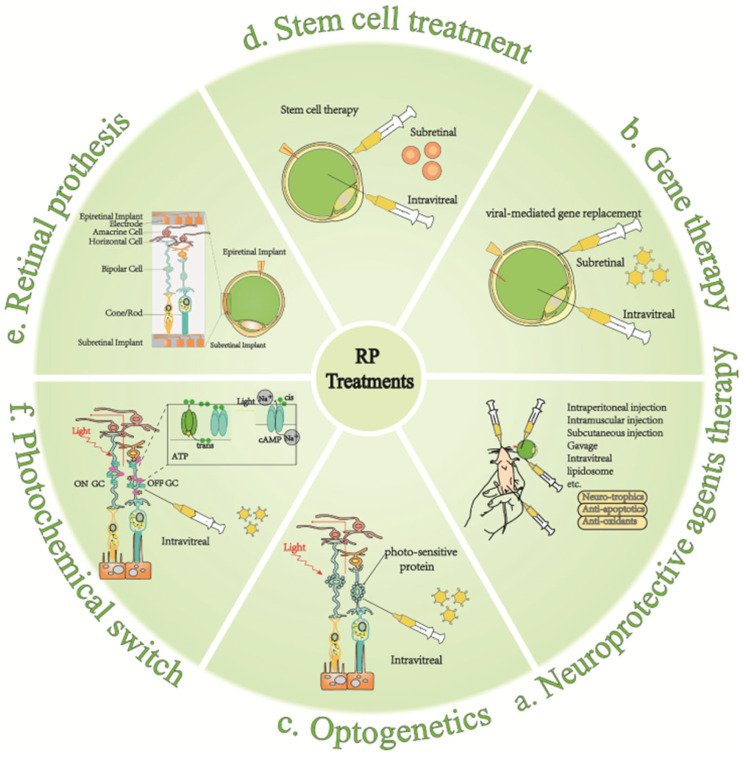
Six current treatment strategies for retinitis pigmentosa (RP). (**a**) Neuroprotective agents mainly include neurotrophic factors, anti-apoptotic agents, and antioxidants; they are usually used in the early stages of the disease and can also serve as the adjunctive treatment in other stages; (**b**) Gene therapy takes effect via the virus-mediated injection of a therapeutic gene tool into the retina in vitro to replace the disease-causing gene; (**c**) Introducing photosensitive optic proteins into the degenerated retina for ectopic expression in damaged cell membranes to restore the cone function and conferring photosensitive ability to residual retinal cells, such as bipolar cells or ganglion cells; (**d**) Injecting neural stem cells cultured in vitro into the retinal injury site induces differentiation into the injured cell type and replacement of injured cells, with the remaining retinal neurons forming synaptic connections; (**e**) Retinal prosthesis implantation at the site of retinal damage; the implantation sites of the artificial retina vary from subretinal, epiretinal membranes to intra-scleral; (**f**) injecting residual non-photoreceptor cells with genes that express ion channel proteins, and attaching “photoswitches”—chemical molecules that change shape when exposed to light—to the ion channel proteins.

Neuroprotective agent therapy is one of the earliest and most widely used approaches, well-tolerated, and with few side effects (Figure 5a). It is usually used in the early stages of the disease and can also serve as the adjunctive treatment in other stages [11]. Neuroprotective agents mainly include neurotrophic factors, anti-apoptotic agents, and antioxidants. Among them, neurotrophic factors include ciliary neurotrophic factor (CNTF) [159,160], brain-derived neurotrophic factor (BDNF) [161,162], and fibroblast growth factor (FGF) [163,164]. CNTF is one of the cytokines with the best efficacy in slowing retinal degeneration. In randomized clinical trials, it exerts neuroprotective effects by upregulating protein hydrolysis inhibitors, which prevent the degradation of intracellular proteins and extracellular matrix material in randomized controlled clinical trials [165]. Taurine deoxycholic acid (TUDCA) has shown potential therapeutic benefits as an anti-apoptotic agent. In many disease models, it reduces endoplasmic reticulum stress and stabilizes the UPR [166,167]. Vitamin A [168,169,170], docosahexaenoic acid (DHA) [171,172], and luteolin [173,174] are antioxidants that have all been proven effective and safe in large-scale, long-term randomized clinical trials; they have potential in RP treatment. Vitamin A converts to retinoic acid (RA) through an oxidative reaction. Physiologically, RA production is dynamically and precisely controlled to maintain normal neuronal development and synaptic plasticity [175,176]. There are other agents with neuroprotective functions, such as retinoids and their derivatives [177], calcium channel blockers [178], calpain inhibitors [179], and valproic acid [180]. Most of them have been tested in clinical trials. Also, strategies to promote the sustained release of these nutrients are being investigated to commence on limitations such as the short half-life of the drugs [160,181]. Table 2 illustrates neuroprotective agents in pharmacological treatment.

### 5.2. Gene Therapy

Many different treatments for degenerative retinal diseases are still in clinical trials (Table 3). Inherited genetic mutations that cause photoreceptor degeneration can be corrected through the use of gene replacement therapy, which is suitable for the treatment of early retinal degeneration. Owing to the advantages of spatial structure and immune privilege in the retina, which greatly reduce ocular immune responses and suppress systemic side effects, it is suitable for gene therapy and is already actively engaged in clinical research (Figure 5b). Typically, a phase-III clinical trial of the therapeutic *Rpe65* gene transduction has been proven to be safe and effective in patients with RP [206]. 

On the one hand, with the continuous advancement of imaging technologies, such as optical coherence tomography (OCT), real-time qualitative and quantitative assessment of retinal changes after gene therapy has been made possible [11,207]. While the heterogeneity of disease-causing genes has hindered the development of generalized gene therapy strategies [80,208,209,210], the conditional gene therapy approach—controlling transgene expression using small molecule-based modulation of abundance or activity, such as the construction of tetracycline/doxycycline (tet/dox)-based trans-transcriptional activation systems and the direct use of so-called destabilizing structural domains (DHFR DD) to regulate the abundance of small molecule proteins—has achieved initial success [13].

**Figure 6:**
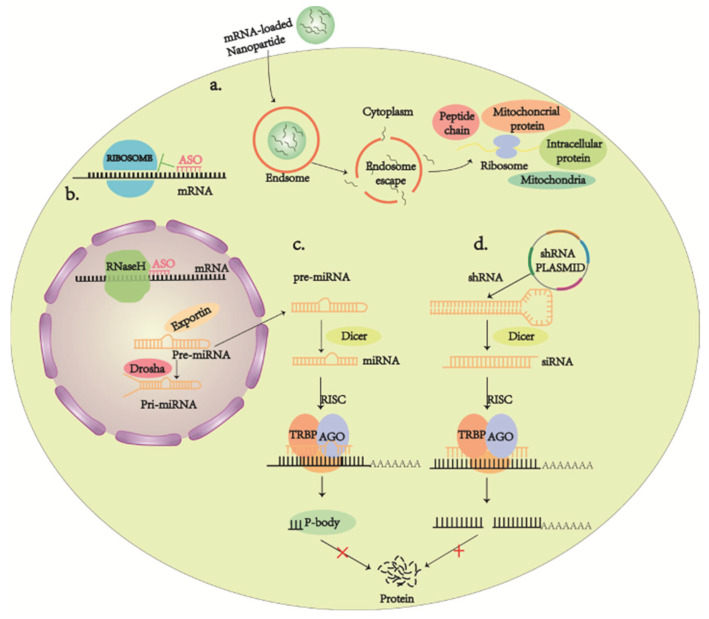
(**a**) In vitro mRNAs delivered into the cytoplasm via special materials are directly translated by ribosomes into various proteins that exert their corresponding effects. (**b**) Antisense oligonucleotides (ASO) as chemically modified short RNA or DNA molecules that bind target mRNAs and can lead to RNase H-induced cleavage (bottom) or inhibit translation (top). (**c**,**d**) RNAi therapies involving small interfering RNAs (SiRNAs) or similar molecules (microRNAs) that are 21–23 nucleotides long to degrade mRNA and prevent its translation into proteins.

On the other hand, RNA therapies, such as nucleases [211,212], RNA interference (RNAi) [213], antisense oligonucleotides (ASO) [214], and mRNA [215], have also been making their way owing to the urgent need to develop alternative therapies, as gene transfer poses quite a few limitations and barriers [216,217,218]. RNA interference (RNAi) is designed to knock down the expression of target genes by binding to target complementary mRNAs, leading to nucleic acid endonuclease-mediated degradation of target mRNAs or the inhibition of mRNA translation (Figure 6c,d). The roles of siRNAs and miRNAs are subtly different from each other in terms of pharmacological practice. miRNA may affect the expression of several different target genes simultaneously, while siRNAs are often able to trigger more effective and specific gene silencing than miRNAs [219]. In 2018, the FDA approved the first RNAi drug, patisiran, to treat the hereditary transport of thyroxine protein amyloidosis [213]. The advantages of ASO over vector-mediated systems are their ease of packaging and their long-term stability for intracellular transport. This means that once ASO enters the cell, it promotes RNA degradation or interferes with mRNA splicing; ASO may also interfere with post-transcriptional mRNA processing or translation (Figure 6a) [214]. Nowadays, mRNA therapeutics is recognized as a new class of drugs. However, it has been hampered by issues such as instability and immunogenicity, making it less desirable than DNA-related gene therapy. However, these critical issues have been resolved, mainly by the introduction of modified nucleosides in mRNA sequences and the development of various RNA packaging and delivery systems in recent years. Much evidence suggests that mRNA not only exerts superior transfection efficiency and a longer protein expression period, but is also economical over DNA [220] (Figure 6b). For example, antibody-functionalized nanostar technology is a method of gene delivery that holds promise for guiding clinical practice in mediating selective RNA therapies [221].

Surprisingly, the emerging CRISPR/Cas9-editing system in recent years has achieved better versatility and precision in facilitating gene correction [222]. Simultaneously, various limitations of this technology are gradually being addressed by developing countermeasures, such as the non-homologous targeted integration (HITI) technique designed to improve the effectiveness of CRISPR/Cas9 [223]. In addition to in vivo gene transfer treatment strategies, the partial restoration of visual function has also been achieved by fusing rAAV2 vectors and MC vectors to first correct mutated genes in photoreceptor cell precursors in vitro and in vivo [224]. Nevertheless, the realization of gene therapy from bench to bedside still faces many challenges, such as the identification and molecular diagnosis of gene mutations, the limitations of gene transfer technology, and the long-term effectiveness and safety, all of which still warrant long-term follow-up, in-depth research, and comprehensive evaluation [216,222].

### 5.3. Stem Cell Therapy

Currently, it appears that gene and cell replacement therapies are most beneficial for patients with retinal degeneration before complete loss of optic rod cells and cone cells (Table 3). Cell transplantation is a cell therapy technique in which normal cells are transferred to replace functionally impaired cells and form synaptic connections with the remaining retinal neurons [225] (Figure 5d). Two main sources of transplanted cells as donors are retinal photoreceptor cell precursors and differentiated embryonic stem cells [181,226]. As transplanted photoreceptor cells only need to establish some short synaptic connections with bipolar cells to transmit electrical signals to downstream pathways, the development of this technology has focused on engineering the generation of functional photoreceptor cells [227]. Since the first three-dimensional culture protocol for generating outer retinal cells from human pluripotent stem cells (hPSCs) [228], hPSCs have been showing promising applications in clinical trials [229]. Meanwhile, ultra-lightweight biodegradable scaffold materials that effectively improve the cellular load have been continuously updated to address all the problems of cell reflux that occur with cell suspension injections [230]. Several clinical trials of cell transplantation for RP have been performed in recent years, which were proven to ameliorate poor vision with no side effects observed yet [231,232,233]. Retinal cells derived from human induced pluripotent stem cells (iPSC) survived in the retinas of photodamaged primate hosts and showed signs of restoration of light response [234]. In animal disease models, adipose-derived stem cells (ASC) showed better protection against degenerated retinas after transplantation into the subretinal space [235]. Besides replacing photoreceptor cells, it is also viable to transplant cells that protect host photoreceptor cells through releasing trophic factors, such as cytokines, in a paracrine manner [236]. Although clinical trials using stem cells have generated a relatively safe profile, many obstacles and concerns, including long-term efficacy, rejection, and inflammation, remain [237]. In addition, the properties of the endoplasmic reticulum (ER) of human pluripotent stem cells (hPSCs) render hPSCs susceptible to stress-induced death [238].

### 5.4. Optogenetics

While gene- and cell-replacement therapies are most beneficial for patients with advanced retinal degeneration, after the complete loss of optic rod cells and cone cells, it is necessary to consider other approaches being developed to treat advanced retinal degeneration. Optogenetics has received increasing attention in recent years, based on its concept of introducing photosensitive optic proteins into the degenerated retina for ectopic expression in damaged cell membranes to restore the cone function and conferring the photosensitive ability to residual retinal cells, such as bipolar cells or ganglion cells [239,240] (Figure 5c). To date, proof of concept has been obtained in animals, and phase-I/II clinical trials are underway [241]. Early studies found that the insertion of algal retinal channelrhodopsin 2 (ChR2) into mammalian cell membranes exhibited a depolarizing effect in response to light [242], which involves a light-driven sodium pump molecular mechanism that can be explained by a specific data model (Panama Canal model) [243,244]. In another study, rod precursors from P4 donor mice were transformed with the photosensitive protein Natronomonas pharaonis halorhodopsin (NpHR); the transformed rods were then isolated and implanted into the retinas of Cpfl1/Rho-/-recipient mice, whose was vision restored afterward [245]. Technological upgrades in optogenetic applications are being achieved through an in-depth understanding of the dynamics, structure, molecular transport modes, and optical properties of photosensitive proteins today [99,246]. Moreover, it remains a great challenge to minimize the adverse effects of introducing photosensitive proteins into photoreceptor cells and activating the specific light wavelengths of opsins. Opsin engineering technologies, such as neural micro-electrodes and Neuronal Communication (NEC), have also made breakthroughs in addressing this shortage [247,248,249,250,251]. They fully considered safety issues, such as reducing the implantation field while ensuring effectiveness [252,253]. Wireless photopoles have been developed in the past to achieve the effect of wireless light stimulation and closed-loop circuitry on real-time control neurons. This technique does not require restraining the animal during the implantation of optical fibers [254,255]. More recently, researchers have succeeded in developing photosensitive proteins with near-infrared (NIR) activation wavelengths, which can reduce light scattering to allow deeper light penetration [256]. Such breakthroughs provide an environment closer to that of the natural retina for the generation of vision stimulated by optogenetic techniques and the precise transmission of neuronal activity.

### 5.5. Artificial Retina

In the advanced stages of retinitis pigmentosa, severe photoreceptor loss occurs. However, the bipolar cells and ganglion cells upstream of the photoreceptors can be sufficiently preserved to convey information to the brain. For legally blind patients suffering from advanced hereditary retinal atrophy with very low vision, this condition can be partially restored using a retinal prosthesis device. The artificial retina was designed using similar principles as the cochlear implant to simulate the function of a human retina that has lost the ability to work [257]. The implantation sites of the artificial retina vary from subretinal, epiretinal membranes to intra-scleral [258] (Figure 5e). To date, only two prostheses (Argus II and Alpha-IMS) have been marketed and have reported long-term follow-up data. For example, the FDA has approved the Argus II retinal prosthesis from EYES for clinical treatment [259,260]. The recently developed 3D visualization system, which is characterized by high resolution, high dynamic range, and increased imaging field depth, has been particularly useful in creating large Argus scleral dissections and during array localization and positioning. Three consecutive cases of successful Argus II implantation using 3D visualization have been reported; no complications or contamination have been found to date [261]. Alpha-IMS (Retina Implant AG, Reutlingen, Germany) is also available commercially. An interim analysis of a clinical trial enrolling 29 patients (including 25 with RP) showed that Alpha-IMS improved daily living activities, as well as light perception and object recognition in 72% and 86% of visually blind participants caused by hereditary retinal degeneration, respectively [262,263]. Post-implantation visual rehabilitation involving training and assessing has also been valued recently, which turned out to improve patients’ adaptation and application ability [264]. Retinal implants, although effective in restoring some visual function in blind patients, face a different set of challenges. For example, these implants require invasive surgery and rely on the extracellular electrical stimulation of the RGC, which can be cytotoxic at high stimulation intensities [265]. The spatial acuity of retinal implants is limited by the number of stimulating electrodes [266]. Moreover, adverse events in early clinical trials, such as retinal ruptures in subjects during implant positioning, conjunctival complications during implant residence, and the disruption of the blood retinal barrier, have led to the discontinuation of some retinal implant clinical trials. All the above examples suggest that more work needs to be conducted to further improve the safety of the device and the method of operation [267].

### 5.6. Chemical Photoswitches

In advanced cases of retinal degeneration, once most or all of the photoreceptors are lost and subsequent retinal remodeling is well underway, general replacement therapies may no longer be beneficial. For example, in advanced cases of retinal degeneration, stem cells may have more difficulty differentiating into the missing cell type and establishing proper synaptic connections. At this point, the later stages of retinal degeneration and retinal remodeling offer some unique opportunities for chemical photoswitch therapy to act primarily on the remaining bipolar cells and/or retinal ganglion cells. Chemical photoswitch therapy usually involves injecting residual non-photoreceptor cells with genes that express ion channel proteins and attaching “photoswitches”—chemical molecules that change shape when exposed to light—to the ion channel proteins. When the photoswitch perceives light stimulation, it opens the ion channels and activates the retinal cells, thereby restoring their photoreceptor properties (Figure 5f). Many different “photoswitches” have been developed to photosensitize cellular proteins (enzymes, neurotransmitter receptors, and ion channels) for reversible regulation controlled by compounds [268,269]. Some photoswitch molecules, such as AAQ (acrylamide–azobenzene–quaternary) and DENAQ (diethylamino–azobenzene–quaternary), block the K^+^ channels to increase non-photosensitive cell excitability with trans conformation. In contrast, it exhibits the opposite effect with cis conformation [270,271]. Because of its biocompatibility [272], the photoswitch is a more natural photo-stimulator and less invasive than retinal prosthesis. However, poor sensitivity to light and possible immune reactions to ectopically expressed proteins are difficulties faced by photoswitch molecules [273]. Researchers have now developed ocularly injectable photoreceptor-binding upconversion nanoparticles (pbUCNPs) that act as light sensors to enable animals to detect both NIR and visible light images. NIR light is projected into the retina through the cornea and lens, the optical portion of the eye; after that, the pbUCNPs convert NIR light into visible light, which activates the photoreceptor cells. It has also been suggested that incorporating pbUCNP into a drug delivery system enables better performation [274]. If applied to a photoswitch, the novel NIR sensor may hold promise for addressing the poor light sensitivity of the photoswitch molecule. One study evaluated the use of two photoswitchable azobenzene ion channel blockers, DAQ and DAA, for vision restoration. While DAQ acts primarily on RGCs, DAA induces light-dependent spiking waves primarily through the activation of amygdala cells. This study found that the degeneration-induced local field potentials remain a major challenge common to all vision restoration approaches [275].

## 6. Summary and Prospects

To date, the field of RP is undergoing a significant paradigm shift with better knowledge and the advancement of scientific research techniques. Especially nowadays, gene therapy is advancing by leaps and bounds, and the corresponding translational application of RP treatment has also achieved positive phase experimental effects. It is more effective, less invasive, and relatively safer in the short term than retinal transplantation. Drug therapy, stem cell therapy, and optogenetic therapy mentioned above meet the needs of maintaining a relatively intact retina with a more stable retinal metabolic environment for the reactivation of damaged neuronal function or transplantation of exogenous cells. Patients with mid- to late-stage RP usually suffer severe retinal damage, which is prevalent during their initial consultation. Years of case studies and experience suggest that all patients with RP and those potentially at risk, as much as possible, should have regular eye examinations for risk assessment and early diagnosis that yield a better prognosis. The innovation of gene therapy technology that modifies the disease-causing gene makes individualized and precise RP treatment possible. Although emerging studies on gene therapy have been on the way to improve vision in patients with RP, they are faced with several bottlenecks, such as burgeoning but immature gene transfer technology and a lack of animal models. Moreover, similar to other therapies, the efficacy, security, and stability of gene therapy warrants further improvement. Nevertheless, as increasing attention is being paid to the disease, with each therapeutic area being actively and positively developing, we have stepped into a new era in the diagnosis and treatment of RP.

## Figures and Tables

**Table 1:** RP clinical evaluation and diagnostic status.

Assessment Items		Diagnosis	Reference
**History**	Ocular (documentation of age and course of onset); Medical; Pedigree (family history);	Establish an initial profile. In general, initial symptoms (night blindness, difficulty with dark adaptation, and loss of mid-peripheral vision) begin in adolescence; the age of onset is highly variable and difficult to determine. Including current and history use of retinotoxic medication Mapping genetic genealogy, assessing inheritance patterns, and identifying potentially diseased members.	[131,132]
**Clinical eye** **examination**	Best-corrected visual acuity: ETDRs (or equivalent); Slit-lamp biomicroscopy; Intraocular pressure; Indirect Ophthalmoscopy;	Identifying ocular features that interfere with vision.	[133,134,135,136,137,138,139]
	Spectral-domain optical coherence tomography (OCT)	Provides cross-sectional imaging of the fibrous layer. The thickness of the outer nuclear layer of the outer segment gradually decreases, with a tubular structure of the outer nuclear layer in the late stages, and decreases in the thickness of the outer nuclear layer; thickening of the inner nuclear layer. Hyperreflective foci commonly found in the inner/outer/inferior space. Helpful in diagnosing macular abnormalities (CME).	[140,141,142,143]
**Retinal imaging**	Fundus imaging Conventional fundus photography; Confocal scanning laser ophthalmoscopy; Multicolor imaging;	With limitations of media opacity and inadequate pupil dilation. Ultra-wide field imaging, but peripheral images are prone to distortion. ith specific wavelengths of reflectance of three lasers to detect information of different layers of the retina and better processing of macular boundary information.	[136,144,145]
	Fundus autofluorescence: Short wavelength (SW)-FAF using blue or green light, with signals originating from lipofuscin. Near-infrared (NIR)-FAF showing an autofluorescent signal originating from the RPE that is less likely to originate from choroidal melanin or related fluorescent groups.	Most FAF cases to evaluate and monitor the progression of RP. 50–60% of patients with RP present with an abnormal foveal ring or autofluorescent ring, ranging from 3 to 20° in diameter, with high interocular symmetry. The ring diameter becomes smaller over time, and the rate of ring reduction varies, with larger rings decreasing more rapidly relative to smaller rings. In the end, the rings disperse. Intra-ring visual sensitivity remains relatively preserved, the ring area itself decreases, and the extra-ring area decreases or cannot be recorded.	[144,145,146,147]
	Fluorescence angiography/optical coherence tomography angiography (OCTA)	Tends to observe choroidal retinal atrophy. (Not commonly used).	[148]
	Adaptive optics scanning laser ophthalmoscopy (AOSLO)	High-resolution imaging modality to detect disease progression and assess the safety and efficacy of treatment. Detect early photoreceptor cell damage (even if the external retinal structures on OCT appear intact). Reveal a decrease in retinal cone cell density prior to a decrease in visual acuity.	[149,150]
**Visual fields** **(VF)**	Kinetic perimetry: assessment of peripheral visual field loss Static perimetry: central visual field loss Fundus-driven Perimetry (microperimetry): Central visual field loss	Record the range of visual function from the center to the far edge.	[151,152,153]
**Electroretin-** **ography**	Full-field ERG^e^ Multifocal ERG^e^	One of the important parameters for the diagnosis and staging of RP. Testing of central and even peripheral optic rod and cone cells for whole retinal functions, such as changes in the a-wave (whether it is lower than normal), changes in oscillatory potential (whether it is reduced), and changes in central cone function (slower decay). Later in the disease process, when whole-field ERG^e^ cannot be tested, multifocal ERGf can still trigger a response.	[154,155,156,157]
**Genetic Diagnostic Testing**	Genetic counseling and targeted treatment Looking for potential new genes		[131,158]

**Table 2:** Neuroprotective agents in pharmacological treatment.

Neuroprotective Agent	Function and Progress	Reference
**CNTF**	Promote rod cell survival;	[182]
	Activate the mTOR pathway of neurons and promote axon regeneration;	[183,184]
	PirB in Müller cells affects RGC neurite regeneration;	[185]
	Upgrading of protein-delivery strategies, such as the novel intravitreal protein delivery strategy CNTF-SH3;	[160,162]
	Neuroinflammatory response induced by CNTF.	[186]
**BDNF**	BDNF inhibits autophagy and promotes synaptic plasticity;	[187]
	BDNF activates protein kinaseC (PKC) to promote synaptic plasticity;	[162]
	Adherent to other therapies: first polymer carrier (non-viral gene delivery) for the delivery of BDNF;	[188]
	Tau neurotoxicity provokes alterations in the BDNF system.	[189]
**FGF**	Non-enzymatic molecular scaffold α-Klotho promotes FGF23 signaling;	[190]
	Participates in GLP-1 receptor signaling to regulate fatty acid oxidation, mitochondrial integrity, and functioning.	[191]
**TUCDCA**	Neuroprotection of retinal neurons by TUDCA; TUDCA affects stem cell survival, proliferation, and transformation;	[192,193,194]
	Epigenetic regulatory activity.	[195,196,197]
**VA**	RGR protein is involved in the light-driven regeneration of cone visual pigments.	[169,170]
**Lutein**	Blue light-filtering characteristics;	[198]
	Antioxidant, anti-inflammatory.	[199]
**DHA**	Induces endogenous antioxidants and mobilizes the selective autophagy of misfolded proteins;	[171]
	Adiponectin receptor 1 conserves docosahexaenoic acid and promotes photoreceptor cell survival.	[172]
**Calcium Channel Blockers**	A novel L-type voltage gate calcium channel blocker and application for the prevention of inflammation and angiogenesis;	[200]
	Structure and pharmacology of voltage-gated sodium and calcium channels.	[201]
**Calpain Inhibitor**	Calpain inhibition spares oligodendrocytes, prevents the degradation of axonal neurofilament protein, and attenuates reactive astrocytosis.	[202]
	Randomized phase-2 multicenter placebo-controlled clinical trial;	[203]
**VPA**	Vitamin C- and valproic acid-induced fetal RPE stem-like cells recover retinal degeneration via regulating SOX2.	[204]
**HDACi**	Rescue cone photoreceptor-mediated visual function.	[205]

**Table 3:** Ongoing clinical trials of potential gene and cell therapies for retinitis pigmentosa (clinicaltrials.gov).

Status	Study Title	Interventions
Gene therapy		
Recruiting (Phase 2)	A First-in-human, Proof of Concept Study of CPK850 in Patients With RLBP1 Retinitis Pigmentosa	Biological: CPK850
Active, not recruiting (Phase 2)	Safety and Efficacy Study in Patients With Retinitis Pigmentosa Due to Mutations in PDE6B Gene	Biological: AAV2/5-hPDE6B
Recruiting (Phase 2)	4D-125 in Patients With X-Linked Retinitis Pigmentosa (XLRP)	Biological: 4D-125 IVT Injection Other: Observational
Recruiting (Phase 3)	Gene Therapy Trial for the Treatment of X-linked Retinitis Pigmentosa Associated With Variants in the RPGR Gene	Biological: Genetic: AAV5-RPGR
Recruiting (Phase 3)	Follow-up Gene Therapy Trial for the Treatment of X-linked Retinitis Pigmentosa Associated With Variants in the RPGR Gene	Biological: Genetic: AAV5-RPGR 4e11 Biological: Genetic: AAV5-RPGR 2e11
Recruiting (Phase 2)	Dose-escalation Study to Evaluate the Safety and Tolerability of GS030 in Subjects With Retinitis Pigmentosa	Combination Product: Gene therapy: GS030-DP AND Medical device: GS030-MD
Not yet recruiting (Phase 3)	A Clinical Trial Evaluating the Safety and Efficacy of a Single Subretinal Injection of AGTC-501 in Participants With X-linked Retinitis Pigmentosa Caused by RPGR Mutations	Biological: rAAV2tYF-GRK1-hRPGRco
Recruiting (Phase 2)	Safety and Efficacy of rAAV2tYF-GRK1-RPGR in Subjects With X-linked Retinitis Pigmentosa Caused by RPGR Mutations	Biological: rAAV2tYF-GRK1-RPGR
Recruiting (Phase 1/2)	Long Term Follow-Up Gene Therapy Study for XLRP RPGR	Biological: AAV-RPGR
Active, not recruiting (Phase 2)	Efficacy and Safety of vMCO-010 Optogenetic Therapy in Adults With Retinitis Pigmentosa [RESTORE]	Biological: Gene therapy product—vMCO-010 Procedure: Sham injection
Cell therapy		
Recruiting (Phase 1)	Pilot Study of Intravitreal Autologous CD34+ Stem Cell Therapy for Retinitis Pigmentosa	Biological: Intravitreal autologous CD34+ cells
Recruiting (Phase 2)	Investigation of Therapeutic Efficacy and Safety of UMSCs for the Management of Retinitis Pigmentosa (RP)	Biological: Injection of stem cells in the sub-tenon space of eye for the management of retinitis pigmentosa Biological: Injection of stem cells in suprachoroidal space of eye for the management of retinitis pigmentosa
Active, not recruiting (Phase 2)	Safety of Repeat Intravitreal Injection of Human Retinal Progenitor Cells (jCell) in Adult Subjects With Retinitis Pigmentosa	Biological: human retinal progenitor cells
Unknown † (Phase 1)	Safety and Efficacy of Subretinal Transplantation of Clinical Human Embryonic Stem Cell Derived Retinal Pigment Epitheliums in Treatment of Retinitis Pigmentosa	Biological: Retinal pigment epitheliums transplantation
Unknown † (Phase 2)	Clinical Study to Evaluate Safety and Efficacy of BMMNC in Retinitis Pigmentosa	Biological: BMMNCs
Recruiting (Phase 1)	CNS10-NPC for the Treatment of RP	Biological: CNS10-NPC implantation
Unknown † (Phase 2)	Autologous Bone Marrow-Derived CD34+, CD133+, and CD271+ Stem Cell Transplantation for Retinitis Pigmentosa	Biological: Stem cell transplantation
Recruiting (Phase 2)	Interventional Study of Implantation of hESC-derived RPE in Patients With RP Due to Monogenic Mutation	Biological: Human embryonic stem cell-derived retinal pigment epithelium (RPE)
Unknown † (Early Phase 1)	Treatment of RP and LCA by Primary RPE Transplantation	Biological: Human primary retinal pigment epithelial (HuRPE) cells
Unknown † (Phase 1)	Stem Cells Therapy in Degenerative Diseases of the Retina	Biological: Stem/progenitor cells transplantation
Recruiting (Phase 1)	Safety of Cultured Allogeneic Adult Umbilical Cord Derived Mesenchymal Stem Cells for Eye Diseases	Biological: AlloRx
Drug treatment		
Recruiting (Phase 2)	PDE6A Gene Therapy for Retinitis Pigmentosa	Drug: Subretinal injection of rAAV.hPDE6A
Recruiting (Phase 1/2)	The Study to Assess the Safety and Efficacy of OCU400 for Retinitis Pigmentosa	Drug: OCU400 Low Dose Drug: OCU400 Mid Dose Drug: OCU400 High Dose
Recruiting (Phase 1/2)	BS01 in Patients With Retinitis Pigmentosa	Drug: BS01

† Study has passed its completion date and status has not been verified in more than two years.

## References

[1] Dias M.F., Joo K., Kemp J.A., Fialho S.L., da Silva Cunha A., Woo S.J., Kwon Y.J. (2018). Molecular genetics and emerging therapies for retinitis pigmentosa: Basic research and clinical perspectives. Prog. Retin. Eye Res..

[2] Tsang S.H., Sharma T. (2018). Retinitis Pigmentosa (Non-syndromic). Adv. Exp. Med. Biol..

[3] Michalakis S., Koch S., Sothilingam V., Garcia Garrido M., Tanimoto N., Schulze E., Becirovic E., Koch F., Seide C., Beck S.C. (2014). Gene therapy restores vision and delays degeneration in the CNGB1(-/-) mouse model of retinitis pigmentosa. Adv. Exp. Med. Biol..

[4] Jordan S.A., Farrar G.J., Kenna P., Humphries M.M., Sheils D.M., Kumar-Singh R., Sharp E.M., Soriano N., Ayuso C., Benitez J. (1993). Localization of an autosomal dominant retinitis pigmentosa gene to chromosome 7q. Nat. Genet..

[5] Banerjee P., Kleyn P.W., Knowles J.A., Lewis C.A., Ross B.M., Parano E., Kovats S.G., Lee J.J., Penchaszadeh G.K., Ott J. (1998). TULP1 mutation in two extended Dominican kindreds with autosomal recessive retinitis pigmentosa. Nat. Genet..

[6] Maw M.A., Kennedy B., Knight A., Bridges R., Roth K.E., Mani E.J., Mukkadan J.K., Nancarrow D., Crabb J.W., Denton M.J. (1997). Mutation of the gene encoding cellular retinaldehyde-binding protein in autosomal recessive retinitis pigmentosa. Nat. Genet..

[7] Vervoort R., Lennon A., Bird A.C., Tulloch B., Axton R., Miano M.G., Meindl A., Meitinger T., Ciccodicola A., Wright A.F. (2000). Mutational hot spot within a new RPGR exon in X-linked retinitis pigmentosa. Nat. Genet..

[8] Bunker C.H., Berson E.L., Bromley W.C., Hayes R.P., Roderick T.H. (1984). Prevalence of retinitis pigmentosa in Maine. Am. J. Ophthalmol..

[9] Hamel C. (2006). Retinitis pigmentosa. Orphanet J. Rare Dis..

[10] Tam B.M., Moritz O.L. (2006). Characterization of rhodopsin P23H-induced retinal degeneration in a Xenopus laevis model of retinitis pigmentosa. Investig. Ophthalmol. Vis. Sci..

[11] Mathur P., Yang J. (2015). Usher syndrome: Hearing loss, retinal degeneration and associated abnormalities. Biochim. Biophys. Acta.

[12] Pearring J.N., Salinas R.Y., Baker S.A., Arshavsky V.Y. (2013). Protein sorting, targeting and trafficking in photoreceptor cells. Prog. Retin. Eye Res..

[13] Swaroop A., Kim D., Forrest D. (2010). Transcriptional regulation of photoreceptor development and homeostasis in the mammalian retina. Nat. Rev. Neurosci..

[14] Krol J., Roska B. (2015). Rods Feed Cones to Keep them Alive. Cell.

[15] van Soest S., Westerveld A., de Jong P.T., Bleeker-Wagemakers E.M., Bergen A.A. (1999). Retinitis pigmentosa: Defined from a molecular point of view. Surv. Ophthalmol..

[16] Slijkerman R.W., Song F., Astuti G.D., Huynen M.A., van Wijk E., Stieger K., Collin R.W. (2015). The pros and cons of vertebrate animal models for functional and therapeutic research on inherited retinal dystrophies. Prog. Retin. Eye Res..

[17] Lakkaraju A., Umapathy A., Tan L.X., Daniele L., Philp N.J., Boesze-Battaglia K., Williams D.S. (2020). The cell biology of the retinal pigment epithelium. Prog. Retin. Eye Res..

[18] Jones B.W., Pfeiffer R.L., Ferrell W.D., Watt C.B., Marmor M., Marc R.E. (2016). Retinal remodeling in human retinitis pigmentosa. Exp. Eye Res..

[19] Murakami Y., Notomi S., Hisatomi T., Nakazawa T., Ishibashi T., Miller J.W., Vavvas D.G. (2013). Photoreceptor cell death and rescue in retinal detachment and degenerations. Prog. Retin. Eye Res..

[20] Susin S.A., Lorenzo H.K., Zamzami N., Marzo I., Snow B.E., Brothers G.M., Mangion J., Jacotot E., Costantini P., Loeffler M. (1999). Molecular characterization of mitochondrial apoptosis-inducing factor. Nature.

[21] Ramirez M.L.G., Salvesen G.S. (2018). A primer on caspase mechanisms. Semin. Cell Dev. Biol..

[22] Peter M.E., Krammer P.H. (2003). The CD95(APO-1/Fas) DISC and beyond. Cell Death Differ..

[23] Micheau O., Tschopp J. (2003). Induction of TNF receptor I-mediated apoptosis via two sequential signaling complexes. Cell.

[24] Horn S., Hughes M.A., Schilling R., Sticht C., Tenev T., Ploesser M., Meier P., Sprick M.R., MacFarlane M., Leverkus M. (2017). Caspase-10 Negatively Regulates Caspase-8-Mediated Cell Death, Switching the Response to CD95L in Favor of NF-κB Activation and Cell Survival. Cell Rep..

[25] Li P., Allen H., Banerjee S., Franklin S., Herzog L., Johnston C., McDowell J., Paskind M., Rodman L., Salfeld J. (1995). Mice deficient in IL-1 beta-converting enzyme are defective in production of mature IL-1 beta and resistant to endotoxic shock. Cell.

[26] Wei M.C., Zong W.X., Cheng E.H., Lindsten T., Panoutsakopoulou V., Ross A.J., Roth K.A., MacGregor G.R., Thompson C.B., Korsmeyer S.J. (2001). Proapoptotic BAX and BAK: A requisite gateway to mitochondrial dysfunction and death. Science.

[27] Lin Y., Ma W., Benchimol S. (2000). Pidd, a new death-domain-containing protein, is induced by p53 and promotes apoptosis. Nat. Genet..

[28] Modjtahedi N., Giordanetto F., Madeo F., Kroemer G. (2006). Apoptosis-inducing factor: Vital and lethal. Trends Cell Biol..

[29] Polster B.M., Basañez G., Etxebarria A., Hardwick J.M., Nicholls D.G. (2005). Calpain I induces cleavage and release of apoptosis-inducing factor from isolated mitochondria. J. Biol. Chem..

[30] Yu S.W., Andrabi S.A., Wang H., Kim N.S., Poirier G.G., Dawson T.M., Dawson V.L. (2006). Apoptosis-inducing factor mediates poly(ADP-ribose) (PAR) polymer-induced cell death. Proc. Natl. Acad. Sci. USA.

[31] Churbanova I.Y., Sevrioukova I.F. (2008). Redox-dependent changes in molecular properties of mitochondrial apoptosis-inducing factor. J. Biol. Chem..

[32] Sanges D., Comitato A., Tammaro R., Marigo V. (2006). Apoptosis in retinal degeneration involves cross-talk between apoptosis-inducing factor (AIF) and caspase-12 and is blocked by calpain inhibitors. Proc. Natl. Acad. Sci. USA.

[33] Fricker M., Tolkovsky A.M., Borutaite V., Coleman M., Brown G.C. (2018). Neuronal Cell Death. Physiol. Rev..

[34] Szamier R.B., Berson E.L. (1977). Retinal ultrastructure in advanced retinitis pigmentosa. Investig. Ophthalmol. Vis. Sci..

[35] Murakami Y., Matsumoto H., Roh M., Suzuki J., Hisatomi T., Ikeda Y., Miller J.W., Vavvas D.G. (2012). Receptor interacting protein kinase mediates necrotic cone but not rod cell death in a mouse model of inherited degeneration. Proc. Natl. Acad. Sci. USA.

[36] Kelley N., Jeltema D., Duan Y., He Y. (2019). The NLRP3 Inflammasome: An Overview of Mechanisms of Activation and Regulation. Int. J. Mol. Sci..

[37] Shu D.Y., Butcher E.R., Saint-Geniez M. (2021). Suppression of PGC-1α Drives Metabolic Dysfunction in TGFβ2-Induced EMT of Retinal Pigment Epithelial Cells. Int. J. Mol. Sci..

[38] Stockwell B.R., Friedmann Angeli J.P., Bayir H., Bush A.I., Conrad M., Dixon S.J., Fulda S., Gascón S., Hatzios S.K., Kagan V.E. (2017). Ferroptosis: A Regulated Cell Death Nexus Linking Metabolism, Redox Biology, and Disease. Cell.

[39] Huang B., Liang J.J., Zhuang X., Chen S.W., Ng T.K., Chen H. (2018). Intravitreal Injection of Hydrogen Peroxide Induces Acute Retinal Degeneration, Apoptosis, and Oxidative Stress in Mice. Oxidative Med. Cell. Longev..

[40] Lu L., Oveson B.C., Jo Y.J., Lauer T.W., Usui S., Komeima K., Xie B., Campochiaro P.A. (2009). Increased expression of glutathione peroxidase 4 strongly protects retina from oxidative damage. Antioxid. Redox Signal..

[41] Ueta T., Inoue T., Furukawa T., Tamaki Y., Nakagawa Y., Imai H., Yanagi Y. (2012). Glutathione peroxidase 4 is required for maturation of photoreceptor cells. J. Biol. Chem..

[42] Sun Y., Zheng Y., Wang C., Liu Y. (2018). Glutathione depletion induces ferroptosis, autophagy, and premature cell senescence in retinal pigment epithelial cells. Cell Death Dis..

[43] Mizushima N., Levine B., Cuervo A.M., Klionsky D.J. (2008). Autophagy fights disease through cellular self-digestion. Nature.

[44] Tsukada M., Ohsumi Y. (1993). Isolation and characterization of autophagy-defective mutants of Saccharomyces cerevisiae. FEBS Lett..

[45] Li W., Zhang L. (2019). Regulation of ATG and Autophagy Initiation. Adv. Exp. Med. Biol..

[46] Ohsumi Y. (2001). Molecular dissection of autophagy: Two ubiquitin-like systems. Nat. Rev. Mol. Cell Biol..

[47] Kirisako T., Ichimura Y., Okada H., Kabeya Y., Mizushima N., Yoshimori T., Ohsumi M., Takao T., Noda T., Ohsumi Y. (2000). The reversible modification regulates the membrane-binding state of Apg8/Aut7 essential for autophagy and the cytoplasm to vacuole targeting pathway. J. Cell Biol..

[48] Ichimura Y., Kirisako T., Takao T., Satomi Y., Shimonishi Y., Ishihara N., Mizushima N., Tanida I., Kominami E., Ohsumi M. (2000). A ubiquitin-like system mediates protein lipidation. Nature.

[49] Kunchithapautham K., Rohrer B. (2007). Apoptosis and autophagy in photoreceptors exposed to oxidative stress. Autophagy.

[50] Sethi C.S., Lewis G.P., Fisher S.K., Leitner W.P., Mann D.L., Luthert P.J., Charteris D.G. (2005). Glial remodeling and neural plasticity in human retinal detachment with proliferative vitreoretinopathy. Investig. Ophthalmol. Vis. Sci..

[51] Roesch K., Stadler M.B., Cepko C.L. (2012). Gene expression changes within Müller glial cells in retinitis pigmentosa. Mol. Vis..

[52] Pfeiffer R.L., Marc R.E., Jones B.W. (2020). Persistent remodeling and neurodegeneration in late-stage retinal degeneration. Prog. Retin. Eye Res..

[53] Gorbatyuk M.S., Knox T., LaVail M.M., Gorbatyuk O.S., Noorwez S.M., Hauswirth W.W., Lin J.H., Muzyczka N., Lewin A.S. (2010). Restoration of visual function in P23H rhodopsin transgenic rats by gene delivery of BiP/Grp78. Proc. Natl. Acad. Sci. USA.

[54] Rasheva V.I., Domingos P.M. (2009). Cellular responses to endoplasmic reticulum stress and apoptosis. Apoptosis.

[55] Oakes S.A., Papa F.R. (2015). The role of endoplasmic reticulum stress in human pathology. Annu. Rev. Pathol..

[56] Illing M.E., Rajan R.S., Bence N.F., Kopito R.R. (2002). A rhodopsin mutant linked to autosomal dominant retinitis pigmentosa is prone to aggregate and interacts with the ubiquitin proteasome system. J. Biol. Chem..

[57] Burré J., Sharma M., Südhof T.C. (2015). Definition of a molecular pathway mediating α-synuclein neurotoxicity. J. Neurosci..

[58] Majd S., Power J.H., Grantham H.J. (2015). Neuronal response in Alzheimer’s and Parkinson’s disease: The effect of toxic proteins on intracellular pathways. BMC Neurosci..

[59] Angelova P.R., Abramov A.Y. (2017). Alpha-synuclein and beta-amyloid—Different targets, same players: Calcium, free radicals and mitochondria in the mechanism of neurodegeneration. Biochem. Biophys. Res. Commun..

[60] Murakami Y., Ishikawa K., Nakao S., Sonoda K.H. (2020). Innate immune response in retinal homeostasis and inflammatory disorders. Prog. Retin. Eye Res..

[61] O’Koren E.G., Mathew R., Saban D.R. (2016). Fate mapping reveals that microglia and recruited monocyte-derived macrophages are definitively distinguishable by phenotype in the retina. Sci. Rep..

[62] McMenamin P.G., Saban D.R., Dando S.J. (2019). Immune cells in the retina and choroid: Two different tissue environments that require different defenses and surveillance. Prog. Retin. Eye Res..

[63] Karlstetter M., Scholz R., Rutar M., Wong W.T., Provis J.M., Langmann T. (2015). Retinal microglia: Just bystander or target for therapy?. Prog. Retin. Eye Res..

[64] Murakami Y., Nakabeppu Y., Sonoda K.H. (2020). Oxidative Stress and Microglial Response in Retinitis Pigmentosa. Int. J. Mol. Sci..

[65] Silverman S.M., Ma W., Wang X., Zhao L., Wong W.T. (2019). C3- and CR3-dependent microglial clearance protects photoreceptors in retinitis pigmentosa. J. Exp. Med..

[66] Micera A., Balzamino B.O., Di Zazzo A., Dinice L., Bonini S., Coassin M. (2020). Biomarkers of Neurodegeneration and Precision Therapy in Retinal Disease. Front. Pharmacol..

[67] Scimone C., Donato L., Alibrandi S., Vadalà M., Giglia G., Sidoti A., D’Angelo R. (2021). N-retinylidene-N-retinylethanolamine adduct induces expression of chronic inflammation cytokines in retinal pigment epithelium cells. Exp. Eye Res..

[68] Lin M.T., Beal M.F. (2006). Mitochondrial dysfunction and oxidative stress in neurodegenerative diseases. Nature.

[69] El-Hattab A.W., Scaglia F. (2016). Mitochondrial cytopathies. Cell Calcium.

[70] Rinaldi C., Donato L., Alibrandi S., Scimone C., D’Angelo R., Sidoti A. (2021). Oxidative Stress and the Neurovascular Unit. Life.

[71] Jang J.Y., Blum A., Liu J., Finkel T. (2018). The role of mitochondria in aging. J. Clin. Investig..

[72] Sinha K., Das J., Pal P.B., Sil P.C. (2013). Oxidative stress: The mitochondria-dependent and mitochondria-independent pathways of apoptosis. Arch. Toxicol..

[73] Moreno M.L., Mérida S., Bosch-Morell F., Miranda M., Villar V.M. (2018). Autophagy Dysfunction and Oxidative Stress, Two Related Mechanisms Implicated in Retinitis Pigmentosa. Front. Physiol..

[74] Quinn M.T., Gauss K.A. (2004). Structure and regulation of the neutrophil respiratory burst oxidase: Comparison with nonphagocyte oxidases. J. Leukoc. Biol..

[75] Usui S., Oveson B.C., Lee S.Y., Jo Y.J., Yoshida T., Miki A., Miki K., Iwase T., Lu L., Campochiaro P.A. (2009). NADPH oxidase plays a central role in cone cell death in retinitis pigmentosa. J. Neurochem..

[76] Nishiguchi K.M., Carvalho L.S., Rizzi M., Powell K., Holthaus S.M., Azam S.A., Duran Y., Ribeiro J., Luhmann U.F., Bainbridge J.W. (2015). Gene therapy restores vision in rd1 mice after removal of a confounding mutation in Gpr179. Nat. Commun..

[77] Yamada H., Yamada E., Hackett S.F., Ozaki H., Okamoto N., Campochiaro P.A. (1999). Hyperoxia causes decreased expression of vascular endothelial growth factor and endothelial cell apoptosis in adult retina. J. Cell Physiol..

[78] Lee S.Y., Usui S., Zafar A.B., Oveson B.C., Jo Y.J., Lu L., Masoudi S., Campochiaro P.A. (2011). N-Acetylcysteine promotes long-term survival of cones in a model of retinitis pigmentosa. J. Cell Physiol..

[79] Yoshida N., Ikeda Y., Notomi S., Ishikawa K., Murakami Y., Hisatomi T., Enaida H., Ishibashi T. (2013). Laboratory evidence of sustained chronic inflammatory reaction in retinitis pigmentosa. Ophthalmology.

[80] Xiong W., MacColl Garfinkel A.E., Li Y., Benowitz L.I., Cepko C.L. (2015). NRF2 promotes neuronal survival in neurodegeneration and acute nerve damage. J. Clin. Investig..

[81] Murakami Y., Ikeda Y., Yoshida N., Notomi S., Hisatomi T., Oka S., De Luca G., Yonemitsu Y., Bignami M., Nakabeppu Y. (2012). MutT homolog-1 attenuates oxidative DNA damage and delays photoreceptor cell death in inherited retinal degeneration. Am. J. Pathol..

[82] Martínez-Fernández de la Cámara C., Salom D., Sequedo M.D., Hervás D., Marín-Lambíes C., Aller E., Jaijo T., Díaz-Llopis M., Millán J.M., Rodrigo R. (2013). Altered antioxidant-oxidant status in the aqueous humor and peripheral blood of patients with retinitis pigmentosa. PLoS ONE.

[83] Matsuzawa-Ishimoto Y., Hwang S., Cadwell K. (2018). Autophagy and Inflammation. Annu. Rev. Immunol..

[84] Filomeni G., De Zio D., Cecconi F. (2015). Oxidative stress and autophagy: The clash between damage and metabolic needs. Cell Death Differ..

[85] Gao Q. (2019). Oxidative Stress and Autophagy. Adv. Exp. Med. Biol..

[86] Vázquez M.C., Balboa E., Alvarez A.R., Zanlungo S. (2012). Oxidative stress: A pathogenic mechanism for Niemann-Pick type C disease. Oxidative Med. Cell. Longev..

[87] Chai P., Ni H., Zhang H., Fan X. (2016). The Evolving Functions of Autophagy in Ocular Health: A Double-edged Sword. Int. J. Biol. Sci..

[88] Cadwell K. (2016). Crosstalk between autophagy and inflammatory signalling pathways: Balancing defence and homeostasis. Nat. Rev. Immunol..

[89] Indo H.P., Hawkins C.L., Nakanishi I., Matsumoto K.I., Matsui H., Suenaga S., Davies M.J., St Clair D.K., Ozawa T., Majima H.J. (2017). Role of Mitochondrial Reactive Oxygen Species in the Activation of Cellular Signals, Molecules, and Function. Handb. Exp. Pharmacol..

[90] Intartaglia D., Giamundo G., Conte I. (2021). Autophagy in the retinal pigment epithelium: A new vision and future challenges. FEBS J..

[91] Venkatesh A., Ma S., Le Y.Z., Hall M.N., Rüegg M.A., Punzo C. (2015). Activated mTORC1 promotes long-term cone survival in retinitis pigmentosa mice. J. Clin. Investig..

[92] Pan M., Yin Y., Wang X., Wang Q., Zhang L., Hu H., Wang C. (2021). Mice deficient in UXT exhibit retinitis pigmentosa-like features via aberrant autophagy activation. Autophagy.

[93] Georgakopoulos-Soares I., Chartoumpekis D.V., Kyriazopoulou V., Zaravinos A. (2020). EMT Factors and Metabolic Pathways in Cancer. Front. Oncol..

[94] Shu D.Y., Butcher E., Saint-Geniez M. (2020). EMT and EndMT: Emerging Roles in Age-Related Macular Degeneration. Int. J. Mol. Sci..

[95] Rosales M.A.B., Shu D.Y., Iacovelli J., Saint-Geniez M. (2019). Loss of PGC-1α in RPE induces mesenchymal transition and promotes retinal degeneration. Life Sci. Alliance.

[96] Chan P., Stolz J., Kohl S., Chiang W.C., Lin J.H. (2016). Endoplasmic reticulum stress in human photoreceptor diseases. Brain Res..

[97] Athanasiou D., Aguilà M., Bevilacqua D., Novoselov S.S., Parfitt D.A., Cheetham M.E. (2013). The cell stress machinery and retinal degeneration. FEBS Lett..

[98] Cideciyan A.V., Sudharsan R., Dufour V.L., Massengill M.T., Iwabe S., Swider M., Lisi B., Sumaroka A., Marinho L.F., Appelbaum T. (2018). Mutation-independent rhodopsin gene therapy by knockdown and replacement with a single AAV vector. Proc. Natl. Acad. Sci. USA.

[99] Deisseroth K., Hegemann P. (2017). The form and function of channelrhodopsin. Science.

[100] Yuan L., Kawada M., Havlioglu N., Tang H., Wu J.Y. (2005). Mutations in PRPF31 inhibit pre-mRNA splicing of rhodopsin gene and cause apoptosis of retinal cells. J. Neurosci..

[101] Schaffert N., Hossbach M., Heintzmann R., Achsel T., Lührmann R. (2004). RNAi knockdown of hPrp31 leads to an accumulation of U4/U6 di-snRNPs in Cajal bodies. EMBO J..

[102] Azizzadeh Pormehr L., Ahmadian S., Daftarian N., Mousavi S.A., Shafiezadeh M. (2020). PRPF31 reduction causes mis-splicing of the phototransduction genes in human organotypic retinal culture. Eur. J. Hum. Genet..

[103] Azizzadeh Pormehr L., Daftarian N., Ahmadian S., Rezaei Kanavi M., Ahmadieh H., Shafiezadeh M. (2018). Human organotypic retinal flat-mount culture (HORFC) as a model for retinitis pigmentosa11. J. Cell. Biochem..

[104] Ajmal M., Khan M.I., Neveling K., Khan Y.M., Azam M., Waheed N.K., Hamel C.P., Ben-Yosef T., De Baere E., Koenekoop R.K. (2014). A missense mutation in the splicing factor gene DHX38 is associated with early-onset retinitis pigmentosa with macular coloboma. J. Med. Genet..

[105] Xu M., Xie Y.A., Abouzeid H., Gordon C.T., Fiorentino A., Sun Z., Lehman A., Osman I.S., Dharmat R., Riveiro-Alvarez R. (2017). Mutations in the Spliceosome Component CWC27 Cause Retinal Degeneration with or without Additional Developmental Anomalies. Am. J. Hum. Genet..

[106] Busetto V., Barbosa I., Basquin J., Marquenet É., Hocq R., Hennion M., Paternina J.A., Namane A., Conti E., Bensaude O. (2020). Structural and functional insights into CWC27/CWC22 heterodimer linking the exon junction complex to spliceosomes. Nucleic Acids Res..

[107] Garancher A., Lin C.Y., Morabito M., Richer W., Rocques N., Larcher M., Bihannic L., Smith K., Miquel C., Leboucher S. (2018). NRL and CRX Define Photoreceptor Identity and Reveal Subgroup-Specific Dependencies in Medulloblastoma. Cancer Cell.

[108] Ng L., Lu A., Swaroop A., Sharlin D.S., Swaroop A., Forrest D. (2011). Two transcription factors can direct three photoreceptor outcomes from rod precursor cells in mouse retinal development. J. Neurosci..

[109] Moore S.M., Skowronska-Krawczyk D., Chao D.L. (2020). Targeting of the NRL Pathway as a Therapeutic Strategy to Treat Retinitis Pigmentosa. J. Clin. Med..

[110] Han S., Chen J., Hua J., Hu X., Jian S., Zheng G., Wang J., Li H., Yang J., Hejtmancik J.F. (2020). MITF protects against oxidative damage-induced retinal degeneration by regulating the NRF2 pathway in the retinal pigment epithelium. Redox Biol..

[111] Westfall J.E., Hoyt C., Liu Q., Hsiao Y.C., Pierce E.A., Page-McCaw P.S., Ferland R.J. (2010). Retinal degeneration and failure of photoreceptor outer segment formation in mice with targeted deletion of the Joubert syndrome gene, Ahi1. J. Neurosci..

[112] Bhogaraju S., Cajanek L., Fort C., Blisnick T., Weber K., Taschner M., Mizuno N., Lamla S., Bastin P., Nigg E.A. (2013). Molecular basis of tubulin transport within the cilium by IFT74 and IFT81. Science.

[113] Kubo T., Brown J.M., Bellve K., Craige B., Craft J.M., Fogarty K., Lechtreck K.F., Witman G.B. (2016). Together, the IFT81 and IFT74 N-termini form the main module for intraflagellar transport of tubulin. J. Cell Sci..

[114] Tebbe L., Kakakhel M., Makia M.S., Al-Ubaidi M.R., Naash M.I. (2020). The Interplay between Peripherin 2 Complex Formation and Degenerative Retinal Diseases. Cells.

[115] Donato L., Abdalla E.M., Scimone C., Alibrandi S., Rinaldi C., Nabil K.M., D’Angelo R., Sidoti A. (2021). Impairments of Photoreceptor Outer Segments Renewal and Phototransduction Due to a Peripherin Rare Haplotype Variant: Insights from Molecular Modeling. Int. J. Mol. Sci..

[116] Clarke G., Goldberg A.F., Vidgen D., Collins L., Ploder L., Schwarz L., Molday L.L., Rossant J., Szél A., Molday R.S. (2000). Rom-1 is required for rod photoreceptor viability and the regulation of disk morphogenesis. Nat. Genet..

[117] Kevany B.M., Tsybovsky Y., Campuzano I.D., Schnier P.D., Engel A., Palczewski K. (2013). Structural and functional analysis of the native peripherin-ROM1 complex isolated from photoreceptor cells. J. Biol. Chem..

[118] Stuck M.W., Conley S.M., Naash M.I. (2016). PRPH2/RDS and ROM-1: Historical context, current views and future considerations. Prog. Retin. Eye Res..

[119] Zulliger R., Conley S.M., Mwoyosvi M.L., Al-Ubaidi M.R., Naash M.I. (2018). Oligomerization of Prph2 and Rom1 is essential for photoreceptor outer segment formation. Hum. Mol. Genet..

[120] Strayve D., Makia M.S., Kakakhel M., Sakthivel H., Conley S.M., Al-Ubaidi M.R., Naash M.I. (2020). ROM1 contributes to phenotypic heterogeneity in PRPH2-associated retinal disease. Hum. Mol. Genet..

[121] Pellikka M., Tanentzapf G., Pinto M., Smith C., McGlade C.J., Ready D.F., Tepass U. (2002). Crumbs, the Drosophila homologue of human CRB1/RP12, is essential for photoreceptor morphogenesis. Nature.

[122] Izaddoost S., Nam S.C., Bhat M.A., Bellen H.J., Choi K.W. (2002). Drosophila Crumbs is a positional cue in photoreceptor adherens junctions and rhabdomeres. Nature.

[123] Alves C.H., Pellissier L.P., Wijnholds J. (2014). The CRB1 and adherens junction complex proteins in retinal development and maintenance. Prog. Retin. Eye Res..

[124] Hollyfield J.G. (1999). Hyaluronan and the functional organization of the interphotoreceptor matrix. Investig. Ophthalmol. Vis. Sci..

[125] Kanan Y., Hoffhines A., Rauhauser A., Murray A., Al-Ubaidi M.R. (2009). Protein tyrosine-O-sulfation in the retina. Exp. Eye Res..

[126] Kam J.H., Weinrich T.W., Shinhmar H., Powner M.B., Roberts N.W., Aboelnour A., Jeffery G. (2019). Fundamental differences in patterns of retinal ageing between primates and mice. Sci. Rep..

[127] Ala-Laurila P., Kolesnikov A.V., Crouch R.K., Tsina E., Shukolyukov S.A., Govardovskii V.I., Koutalos Y., Wiggert B., Estevez M.E., Cornwall M.C. (2006). Visual cycle: Dependence of retinol production and removal on photoproduct decay and cell morphology. J. Gen. Physiol..

[128] Bermúdez V., Tenconi P.E., Giusto N.M., Mateos M.V. (2019). Lipid Signaling in Retinal Pigment Epithelium Cells Exposed to Inflammatory and Oxidative Stress Conditions. Molecular Mechanisms Underlying Degenerative Retinal Diseases. Adv. Exp. Med. Biol..

[129] Isken A., Golczak M., Oberhauser V., Hunzelmann S., Driever W., Imanishi Y., Palczewski K., von Lintig J. (2008). RBP4 disrupts vitamin A uptake homeostasis in a STRA6-deficient animal model for Matthew-Wood syndrome. Cell Metab..

[130] Gauthier M.E., Roy S., Cantin L., Salesse C. (2019). Comparison between the enzymatic activity, structure and substrate binding of mouse and human lecithin retinol acyltransferase. Biochem. Biophys. Res. Commun..

[131] Branham K., Yashar B.M. (2013). Providing comprehensive genetic-based ophthalmic care. Clin. Genet..

[132] Sergott R.C. (2014). Retinal segmentation using multicolor laser imaging. J. Neuroophthalmol..

[133] Li Z.Y., Possin D.E., Milam A.H. (1995). Histopathology of bone spicule pigmentation in retinitis pigmentosa. Ophthalmology.

[134] Cellini M., Strobbe E., Gizzi C., Campos E.C. (2010). ET-1 plasma levels and ocular blood flow in retinitis pigmentosa. Can. J. Physiol. Pharmacol..

[135] Hwang Y.H., Kim S.W., Kim Y.Y., Na J.H., Kim H.K., Sohn Y.H. (2012). Optic nerve head, retinal nerve fiber layer, and macular thickness measurements in young patients with retinitis pigmentosa. Curr. Eye Res..

[136] Strong S., Liew G., Michaelides M. (2017). Retinitis pigmentosa-associated cystoid macular oedema: Pathogenesis and avenues of intervention. Br. J. Ophthalmol..

[137] Chebil A., Touati S., Maamouri R., Kort F., El Matri L. (2016). Spectral Domain optical coherence tomography findings in patients with retinitis pigmentosa. Tunis. Med..

[138] Yoshida N., Ikeda Y., Murakami Y., Nakatake S., Tachibana T., Notomi S., Hisatomi T., Ishibashi T. (2015). Vitreous cysts in patients with retinitis pigmentosa. Jpn. J. Ophthalmol..

[139] Grover S., Fishman G.A., Brown J. (1997). Frequency of optic disc or parapapillary nerve fiber layer drusen in retinitis pigmentosa. Ophthalmology.

[140] Liu G., Du Q., Keyal K., Wang F. (2017). Morphologic characteristics and clinical significance of the macular-sparing area in patients with retinitis pigmentosa as revealed by multicolor imaging. Exp. Ther. Med..

[141] Hood D.C., Lazow M.A., Locke K.G., Greenstein V.C., Birch D.G. (2011). The transition zone between healthy and diseased retina in patients with retinitis pigmentosa. Investig. Ophthalmol. Vis. Sci..

[142] Goldberg N.R., Greenberg J.P., Laud K., Tsang S., Freund K.B. (2013). Outer retinal tubulation in degenerative retinal disorders. Retina.

[143] Kuroda M., Hirami Y., Hata M., Mandai M., Takahashi M., Kurimoto Y. (2014). Intraretinal hyperreflective foci on spectral-domain optical coherence tomographic images of patients with retinitis pigmentosa. Clin. Ophthalmol..

[144] Delori F.C., Dorey C.K., Staurenghi G., Arend O., Goger D.G., Weiter J.J. (1995). In vivo fluorescence of the ocular fundus exhibits retinal pigment epithelium lipofuscin characteristics. Investig. Ophthalmol. Vis. Sci..

[145] Keilhauer C.N., Delori F.C. (2006). Near-infrared autofluorescence imaging of the fundus: Visualization of ocular melanin. Investig. Ophthalmol. Vis. Sci..

[146] Teussink M.M., Lambertus S., de Mul F.F., Rozanowska M.B., Hoyng C.B., Klevering B.J., Theelen T. (2017). Lipofuscin-associated photo-oxidative stress during fundus autofluorescence imaging. PLoS ONE.

[147] Tsang S.H., Sharma T. (2018). Fundus Autofluorescence. Adv. Exp. Med. Biol..

[148] Kashani A.H., Chen C.L., Gahm J.K., Zheng F., Richter G.M., Rosenfeld P.J., Shi Y., Wang R.K. (2017). Optical coherence tomography angiography: A comprehensive review of current methods and clinical applications. Prog. Retin. Eye Res..

[149] Georgiou M., Kalitzeos A., Patterson E.J., Dubra A., Carroll J., Michaelides M. (2018). Adaptive optics imaging of inherited retinal diseases. Br. J. Ophthalmol..

[150] Ratnam K., Carroll J., Porco T.C., Duncan J.L., Roorda A. (2013). Relationship between foveal cone structure and clinical measures of visual function in patients with inherited retinal degenerations. Investig. Ophthalmol. Vis. Sci..

[151] Grover S., Fishman G.A., Brown J. (1998). Patterns of visual field progression in patients with retinitis pigmentosa. Ophthalmology.

[152] Talib M., van Schooneveld M.J., van Genderen M.M., Wijnholds J., Florijn R.J., Ten Brink J.B., Schalij-Delfos N.E., Dagnelie G., Cremers F.P.M., Wolterbeek R. (2017). Genotypic and Phenotypic Characteristics of CRB1-Associated Retinal Dystrophies: A Long-Term Follow-up Study. Ophthalmology.

[153] Berson E.L., Rosner B., Weigel-DiFranco C., Dryja T.P., Sandberg M.A. (2002). Disease progression in patients with dominant retinitis pigmentosa and rhodopsin mutations. Investig. Ophthalmol. Vis. Sci..

[154] McCulloch D.L., Marmor M.F., Brigell M.G., Hamilton R., Holder G.E., Tzekov R., Bach M. (2015). ISCEV Standard for full-field clinical electroretinography (2015 update). Doc. Ophthalmol..

[155] Berson E.L. (1981). Retinitis pigmentosa and allied diseases: Applications of electroretinographic testing. Int. Ophthalmol..

[156] Berson E.L. (1993). Retinitis pigmentosa. The Friedenwald Lecture. Investig. Ophthalmol. Vis. Sci..

[157] Messias K., Jägle H., Saran R., Ruppert A.D., Siqueira R., Jorge R., Messias A. (2013). Psychophysically determined full-field stimulus thresholds (FST) in retinitis pigmentosa: Relationships with electroretinography and visual field outcomes. Doc. Ophthalmol..

[158] Branham K., Schlegel D., Fahim A.T., Jayasundera K.T. (2020). Genetic testing for inherited retinal degenerations: Triumphs and tribulations. Am. J. Med. Genet. C Semin. Med. Genet..

[159] Buch P.K., MacLaren R.E., Ali R.R. (2007). Neuroprotective gene therapy for the treatment of inherited retinal degeneration. Curr. Gene Ther..

[160] Delplace V., Ortin-Martinez A., Tsai E.L.S., Amin A.N., Wallace V., Shoichet M.S. (2019). Controlled release strategy designed for intravitreal protein delivery to the retina. J. Control. Release.

[161] Gupta V.K., You Y., Gupta V.B., Klistorner A., Graham S.L. (2013). TrkB receptor signalling: Implications in neurodegenerative, psychiatric and proliferative disorders. Int. J. Mol. Sci..

[162] Jiang X.C., Xiang J.J., Wu H.H., Zhang T.Y., Zhang D.P., Xu Q.H., Huang X.L., Kong X.L., Sun J.H., Hu Y.L. (2019). Neural Stem Cells Transfected with Reactive Oxygen Species-Responsive Polyplexes for Effective Treatment of Ischemic Stroke. Adv. Mater..

[163] Beenken A., Mohammadi M. (2009). The FGF family: Biology, pathophysiology and therapy. Nat. Rev. Drug Discov..

[164] Hecht R., Li Y.S., Sun J., Belouski E., Hall M., Hager T., Yie J., Wang W., Winters D., Smith S. (2012). Rationale-Based Engineering of a Potent Long-Acting FGF21 Analog for the Treatment of Type 2 Diabetes. PLoS ONE.

[165] Lipinski D.M., Barnard A.R., Singh M.S., Martin C., Lee E.J., Davies W.I.L., MacLaren R.E. (2015). CNTF Gene Therapy Confers Lifelong Neuroprotection in a Mouse Model of Human Retinitis Pigmentosa. Mol. Ther..

[166] Omura T., Asari M., Yamamoto J., Oka K., Hoshina C., Maseda C., Awaya T., Tasaki Y., Shiono H., Yonezawa A. (2013). Sodium tauroursodeoxycholate prevents paraquat-induced cell death by suppressing endoplasmic reticulum stress responses in human lung epithelial A549 cells. Biochem. Biophys. Res. Commun..

[167] Fernández-Sánchez L., Lax P., Pinilla I., Martín-Nieto J., Cuenca N. (2011). Tauroursodeoxycholic acid prevents retinal degeneration in transgenic P23H rats. Investig. Ophthalmol. Vis. Sci..

[168] Guadagni V., Novelli E., Piano I., Gargini C., Strettoi E. (2015). Pharmacological approaches to retinitis pigmentosa: A laboratory perspective. Prog. Retin. Eye Res..

[169] Morshedian A., Kaylor J.J., Ng S.Y., Tsan A., Frederiksen R., Xu T., Yuan L., Sampath A.P., Radu R.A., Fain G.L. (2019). Light-Driven Regeneration of Cone Visual Pigments through a Mechanism Involving RGR Opsin in Müller Glial Cells. Neuron.

[170] Choi E.H., Daruwalla A., Suh S., Leinonen H., Palczewski K. (2021). Retinoids in the visual cycle: Role of the retinal G protein-coupled receptor. J. Lipid Res..

[171] Johansson I., Monsen V.T., Pettersen K., Mildenberger J., Misund K., Kaarniranta K., Schønberg S., Bjørkøy G. (2015). The marine n-3 PUFA DHA evokes cytoprotection against oxidative stress and protein misfolding by inducing autophagy and NFE2L2 in human retinal pigment epithelial cells. Autophagy.

[172] Rice D.S., Calandria J.M., Gordon W.C., Jun B., Zhou Y., Gelfman C.M., Li S., Jin M., Knott E.J., Chang B. (2015). Adiponectin receptor 1 conserves docosahexaenoic acid and promotes photoreceptor cell survival. Nat. Commun..

[173] Kijlstra A., Tian Y., Kelly E.R., Berendschot T.T. (2012). Lutein: More than just a filter for blue light. Prog. Retin. Eye Res..

[174] Shyam R., Gorusupudi A., Nelson K., Horvath M.P., Bernstein P.S. (2017). RPE65 has an additional function as the lutein to meso-zeaxanthin isomerase in the vertebrate eye. Proc. Natl. Acad. Sci. USA.

[175] Cunningham T.J., Duester G. (2015). Mechanisms of retinoic acid signalling and its roles in organ and limb development. Nat. Rev. Mol. Cell Biol..

[176] Chen L., Lau A.G., Sarti F. (2014). Synaptic retinoic acid signaling and homeostatic synaptic plasticity. Neuropharmacology.

[177] Ozaki T., Nakazawa M., Kudo T., Hirano S., Suzuki K., Ishiguro S. (2014). Protection of cone photoreceptor M-opsin degradation with 9-cis-β-carotene-rich alga Dunaliella bardawil in Rpe65(-/-) mouse retinal explant culture. Curr. Eye Res..

[178] Pawlyk B.S., Li T., Scimeca M.S., Sandberg M.A., Berson E.L. (2002). Absence of photoreceptor rescue with D-cis-diltiazem in the rd mouse. Investig. Ophthalmol. Vis. Sci..

[179] Yang J., Weimer R.M., Kallop D., Olsen O., Wu Z., Renier N., Uryu K., Tessier-Lavigne M. (2013). Regulation of axon degeneration after injury and in development by the endogenous calpain inhibitor calpastatin. Neuron.

[180] Yamamoto S., Sugawara T., Murakami A., Nakazawa M., Nao I.N., Machida S., Wada Y., Mashima Y., Myake Y. (2012). Topical isopropyl unoprostone for retinitis pigmentosa: Microperimetric results of the phase 2 clinical study. Ophthalmol. Ther..

[181] Jayakody S.A., Gonzalez-Cordero A., Ali R.R., Pearson R.A. (2015). Cellular strategies for retinal repair by photoreceptor replacement. Prog. Retin. Eye Res..

[182] Wen R., Tao W., Li Y., Sieving P.A. (2012). CNTF and retina. Prog. Retin. Eye Res..

[183] Bei F., Lee H.H.C., Liu X., Gunner G., Jin H., Ma L., Wang C., Hou L., Hensch T.K., Frank E. (2016). Restoration of Visual Function by Enhancing Conduction in Regenerated Axons. Cell.

[184] Eriksen A.Z., Eliasen R., Oswald J., Kempen P.J., Melander F., Andresen T.L., Young M., Baranov P., Urquhart A.J. (2018). Multifarious Biologic Loaded Liposomes that Stimulate the Mammalian Target of Rapamycin Signaling Pathway Show Retina Neuroprotection after Retina Damage. ACS Nano.

[185] Yuan R., Yang M., Fan W., Lan J., Zhou Y.G. (2020). Paired Immunoglobulin-like Receptor B Inhibition in Müller Cells Promotes Neurite Regeneration After Retinal Ganglion Cell Injury in vitro. Neurosci. Bull..

[186] Hu Z., Deng N., Liu K., Zhou N., Sun Y., Zeng W. (2020). CNTF-STAT3-IL-6 Axis Mediates Neuroinflammatory Cascade across Schwann Cell-Neuron-Microglia. Cell Rep..

[187] Nikoletopoulou V., Sidiropoulou K., Kallergi E., Dalezios Y., Tavernarakis N. (2017). Modulation of Autophagy by BDNF Underlies Synaptic Plasticity. Cell Metab..

[188] Colgan L.A., Hu M., Misler J.A., Parra-Bueno P., Moran C.M., Leitges M., Yasuda R. (2018). PKCα integrates spatiotemporally distinct Ca^2+^ and autocrine BDNF signaling to facilitate synaptic plasticity. Nat. Neurosci..

[189] Barbereau C., Yehya A., Silhol M., Cubedo N., Verdier J.M., Maurice T., Rossel M. (2020). Neuroprotective brain-derived neurotrophic factor signaling in the TAU-P301L tauopathy zebrafish model. Pharmacol. Res..

[190] Chen G., Liu Y., Goetz R., Fu L., Jayaraman S., Hu M.C., Moe O.W., Liang G., Li X., Mohammadi M. (2018). α-Klotho is a non-enzymatic molecular scaffold for FGF23 hormone signalling. Nature.

[191] Timper K., Del Río-Martín A., Cremer A.L., Bremser S., Alber J., Giavalisco P., Varela L., Heilinger C., Nolte H., Trifunovic A. (2020). GLP-1 Receptor Signaling in Astrocytes Regulates Fatty Acid Oxidation, Mitochondrial Integrity, and Function. Cell Metab.

[192] Froger N., Moutsimilli L., Cadetti L., Jammoul F., Wang Q.P., Fan Y., Gaucher D., Rosolen S.G., Neveux N., Cynober L. (2014). Taurine: The comeback of a neutraceutical in the prevention of retinal degenerations. Prog. Retin. Eye Res..

[193] Jung E.M., Yoo Y.M., Park S.Y., Ahn C., Jeon B.H., Hong E.J., Kim W.Y., Jeung E.B. (2020). Calbindin-D(9k) is a Novel Risk Gene for Neurodegenerative Disease. Cell Physiol. Biochem..

[194] Afşar E., Kırımlıoglu E., Çeker T., Yılmaz Ç., Demir N., Aslan M. (2020). Effect of ER stress on sphingolipid levels and apoptotic pathways in retinal pigment epithelial cells. Redox Biol..

[195] Yoon Y.M., Lee J.H., Yun S.P., Han Y.S., Yun C.W., Lee H.J., Noh H., Lee S.J., Han H.J., Lee S.H. (2016). Tauroursodeoxycholic acid reduces ER stress by regulating of Akt-dependent cellular prion protein. Sci. Rep..

[196] Soares R., Ribeiro F.F., Xapelli S., Genebra T., Ribeiro M.F., Sebastião A.M., Rodrigues C.M.P., Solá S. (2018). Tauroursodeoxycholic Acid Enhances Mitochondrial Biogenesis, Neural Stem Cell Pool, and Early Neurogenesis in Adult Rats. Mol. Neurobiol..

[197] Zhang Y., Qu P., Ma X., Qiao F., Ma Y., Qing S., Zhang Y., Wang Y., Cui W. (2018). Tauroursodeoxycholic acid (TUDCA) alleviates endoplasmic reticulum stress of nuclear donor cells under serum starvation. PLoS ONE.

[198] Junghans A., Sies H., Stahl W. (2001). Macular pigments lutein and zeaxanthin as blue light filters studied in liposomes. Arch. Biochem. Biophys..

[199] Chucair A.J., Rotstein N.P., Sangiovanni J.P., During A., Chew E.Y., Politi L.E. (2007). Lutein and zeaxanthin protect photoreceptors from apoptosis induced by oxidative stress: Relation with docosahexaenoic acid. Investig. Ophthalmol. Vis. Sci..

[200] Saddala M.S., Lennikov A., Mukwaya A., Yang Y., Hill M.A., Lagali N., Huang H. (2020). Discovery of novel L-type voltage-gated calcium channel blockers and application for the prevention of inflammation and angiogenesis. J. Neuroinflamm..

[201] Catterall W.A., Lenaeus M.J., Gamal El-Din T.M. (2020). Structure and Pharmacology of Voltage-Gated Sodium and Calcium Channels. Annu. Rev. Pharmacol. Toxicol..

[202] Smith A.W., Rohrer B., Wheless L., Samantaray S., Ray S.K., Inoue J., Azuma M., Banik N.L. (2016). Calpain inhibition reduces structural and functional impairment of retinal ganglion cells in experimental optic neuritis. J. Neurochem..

[203] Birch D.G., Bernstein P.S., Iannacone A., Pennesi M.E., Lam B.L., Heckenlively J., Csaky K., Hartnett M.E., Winthrop K.L., Jayasundera T. (2018). Effect of Oral Valproic Acid vs Placebo for Vision Loss in Patients With Autosomal Dominant Retinitis Pigmentosa: A Randomized Phase 2 Multicenter Placebo-Controlled Clinical Trial. JAMA Ophthalmol..

[204] Shen H., Ding C., Yuan S., Pan T., Li D., Li H., Huang B., Liu Q. (2020). Vitamin C- and Valproic Acid-Induced Fetal RPE Stem-like Cells Recover Retinal Degeneration via Regulating SOX2. Mol. Ther..

[205] Samardzija M., Masarini K., Ueffing M., Trifunović D. (2019). HDAC Inhibition Prevents Primary Cone Degeneration Even After the Onset of Degeneration. Adv. Exp. Med. Biol..

[206] Ducloyer J.B., Le Meur G., Cronin T., Adjali O., Weber M. (2020). Gene therapy for retinitis pigmentosa. Med. Sci..

[207] Dalkara D., Goureau O., Marazova K., Sahel J.A. (2016). Let There Be Light: Gene and Cell Therapy for Blindness. Hum. Gene Ther..

[208] Dalkara D., Sahel J.A. (2014). Gene therapy for inherited retinal degenerations. Comptes Rendus. Biol..

[209] Fahim A. (2018). Retinitis pigmentosa: Recent advances and future directions in diagnosis and management. Curr. Opin. Pediatr..

[210] Vu K.T., Hulleman J.D. (2017). An inducible form of Nrf2 confers enhanced protection against acute oxidative stresses in RPE cells. Exp. Eye Res..

[211] Yau E.H., Butler M.C., Sullivan J.M. (2016). A cellular high-throughput screening approach for therapeutic trans-cleaving ribozymes and RNAi against arbitrary mRNA disease targets. Exp. Eye Res..

[212] Lewin A.S., Drenser K.A., Hauswirth W.W., Nishikawa S., Yasumura D., Flannery J.G., LaVail M.M. (1998). Ribozyme rescue of photoreceptor cells in a transgenic rat model of autosomal dominant retinitis pigmentosa. Nat. Med..

[213] Adams D., Gonzalez-Duarte A., O’Riordan W.D., Yang C.C., Ueda M., Kristen A.V., Tournev I., Schmidt H.H., Coelho T., Berk J.L. (2018). Patisiran, an RNAi Therapeutic, for Hereditary Transthyretin Amyloidosis. N. Engl. J. Med..

[214] Rossor A.M., Reilly M.M., Sleigh J.N. (2018). Antisense oligonucleotides and other genetic therapies made simple. Pract. Neurol..

[215] Schlake T., Thran M., Fiedler K., Heidenreich R., Petsch B., Fotin-Mleczek M. (2019). mRNA: A Novel Avenue to Antibody Therapy?. Mol. Ther..

[216] Lee J.H., Wang J.H., Chen J., Li F., Edwards T.L., Hewitt A.W., Liu G.S. (2019). Gene therapy for visual loss: Opportunities and concerns. Prog. Retin. Eye Res..

[217] Carvalho L.S., Vandenberghe L.H. (2015). Promising and delivering gene therapies for vision loss. Vision Res..

[218] Liu Q., Collin R.W., Cremers F.P., den Hollander A.I., van den Born L.I., Pierce E.A. (2012). Expression of wild-type Rp1 protein in Rp1 knock-in mice rescues the retinal degeneration phenotype. PLoS ONE.

[219] Hu B., Zhong L., Weng Y., Peng L., Huang Y., Zhao Y., Liang X.J. (2020). Therapeutic siRNA: State of the art. Signal Transduct. Target. Ther..

[220] Weng Y., Li C., Yang T., Hu B., Zhang M., Guo S., Xiao H., Liang X.J., Huang Y. (2020). The challenge and prospect of mRNA therapeutics landscape. Biotechnol. Adv..

[221] Hasanzadeh Kafshgari M., Agiotis L., Largillière I., Patskovsky S., Meunier M. (2021). Antibody-Functionalized Gold Nanostar-Mediated On-Resonance Picosecond Laser Optoporation for Targeted Delivery of RNA Therapeutics. Small.

[222] Trapani I., Auricchio A. (2018). Seeing the Light after 25 Years of Retinal Gene Therapy. Trends Mol. Med..

[223] Suzuki K., Tsunekawa Y., Hernandez-Benitez R., Wu J., Zhu J., Kim E.J., Hatanaka F., Yamamoto M., Araoka T., Li Z. (2016). In vivo genome editing via CRISPR/Cas9 mediated homology-independent targeted integration. Nature.

[224] Barnea-Cramer A.O., Singh M., Fischer D., De Silva S., McClements M.E., Barnard A.R., MacLaren R.E. (2020). Repair of Retinal Degeneration following Ex Vivo Minicircle DNA Gene Therapy and Transplantation of Corrected Photoreceptor Progenitors. Mol. Ther..

[225] Jones M.K., Lu B., Girman S., Wang S. (2017). Cell-based therapeutic strategies for replacement and preservation in retinal degenerative diseases. Prog. Retin. Eye Res..

[226] Mead B., Berry M., Logan A., Scott R.A., Leadbeater W., Scheven B.A. (2015). Stem cell treatment of degenerative eye disease. Stem. Cell Res..

[227] Goureau O., Orieux G. (2020). [Photoreceptor cell transplantation for future treatment of retinitis pigmentosa]. Med. Sci..

[228] Meyer J.S., Shearer R.L., Capowski E.E., Wright L.S., Wallace K.A., McMillan E.L., Zhang S.C., Gamm D.M. (2009). Modeling early retinal development with human embryonic and induced pluripotent stem cells. Proc. Natl. Acad. Sci. USA.

[229] Zarbin M. (2019). Cell-Based Therapy for Retinal Disease: The New Frontier. Methods Mol. Biol..

[230] Lee I.K., Ludwig A.L., Phillips M.J., Lee J., Xie R., Sajdak B.S., Jager L.D., Gong S., Gamm D.M., Ma Z. (2021). Ultrathin micromolded 3D scaffolds for high-density photoreceptor layer reconstruction. Sci. Adv..

[231] Park S.S., Bauer G., Abedi M., Pontow S., Panorgias A., Jonnal R., Zawadzki R.J., Werner J.S., Nolta J. (2014). Intravitreal autologous bone marrow CD34+ cell therapy for ischemic and degenerative retinal disorders: Preliminary phase 1 clinical trial findings. Investig. Ophthalmol. Vis. Sci..

[232] Siqueira R.C., Messias A., Messias K., Arcieri R.S., Ruiz M.A., Souza N.F., Martins L.C., Jorge R. (2015). Quality of life in patients with retinitis pigmentosa submitted to intravitreal use of bone marrow-derived stem cells (Reticell-clinical trial). Stem Cell Res. Ther..

[233] Kuriyan A.E., Albini T.A., Townsend J.H., Rodriguez M., Pandya H.K., Leonard R.E., Parrott M.B., Rosenfeld P.J., Flynn H.W., Goldberg J.L. (2017). Vision Loss after Intravitreal Injection of Autologous “Stem Cells” for AMD. N. Engl. J. Med..

[234] Tu H.Y., Watanabe T., Shirai H., Yamasaki S., Kinoshita M., Matsushita K., Hashiguchi T., Onoe H., Matsuyama T., Kuwahara A. (2019). Medium- to long-term survival and functional examination of human iPSC-derived retinas in rat and primate models of retinal degeneration. EBioMedicine.

[235] Hu C., La H., Wei X., Zhou Y., Ou Q., Chen Z., Zhu X., Xu J.Y., Jin C., Gao F. (2020). Transplantation Site Affects the Outcomes of Adipose-Derived Stem Cell-Based Therapy for Retinal Degeneration. Stem Cells Int..

[236] Cuenca N., Fernández-Sánchez L., McGill T.J., Lu B., Wang S., Lund R., Huhn S., Capela A. (2013). Phagocytosis of photoreceptor outer segments by transplanted human neural stem cells as a neuroprotective mechanism in retinal degeneration. Investig. Ophthalmol. Vis. Sci..

[237] Sugita S., Mandai M., Kamao H., Takahashi M. (2021). Immunological aspects of RPE cell transplantation. Prog. Retin. Eye Res..

[238] Ha T.W., Jeong J.H., Shin H., Kim H.K., Im J.S., Song B.H., Hanna J., Oh J.S., Woo D.H., Han J. (2020). Characterization of Endoplasmic Reticulum (ER) in Human Pluripotent Stem Cells Revealed Increased Susceptibility to Cell Death upon ER Stress. Cells.

[239] Stern J.H., Tian Y., Funderburgh J., Pellegrini G., Zhang K., Goldberg J.L., Ali R.R., Young M., Xie Y., Temple S. (2018). Regenerating Eye Tissues to Preserve and Restore Vision. Cell Stem Cell.

[240] Cehajic-Kapetanovic J., Eleftheriou C., Allen A.E., Milosavljevic N., Pienaar A., Bedford R., Davis K.E., Bishop P.N., Lucas R.J. (2015). Restoration of Vision with Ectopic Expression of Human Rod Opsin. Curr. Biol..

[241] Simunovic M.P., Shen W., Lin J.Y., Protti D.A., Lisowski L., Gillies M.C. (2019). Optogenetic approaches to vision restoration. Exp. Eye Res..

[242] Nagel G., Szellas T., Huhn W., Kateriya S., Adeishvili N., Berthold P., Ollig D., Hegemann P., Bamberg E. (2003). Channelrhodopsin-2, a directly light-gated cation-selective membrane channel. Proc. Natl. Acad. Sci. USA.

[243] Gushchin I., Shevchenko V., Polovinkin V., Borshchevskiy V., Buslaev P., Bamberg E., Gordeliy V. (2016). Structure of the light-driven sodium pump KR2 and its implications for optogenetics. FEBS J..

[244] Kandori H., Inoue K., Tsunoda S.P. (2018). Light-Driven Sodium-Pumping Rhodopsin: A New Concept of Active Transport. Chem. Rev..

[245] Garita-Hernandez M., Lampič M., Chaffiol A., Guibbal L., Routet F., Santos-Ferreira T., Gasparini S., Borsch O., Gagliardi G., Reichman S. (2019). Restoration of visual function by transplantation of optogenetically engineered photoreceptors. Nat. Commun..

[246] Ordaz J.D., Wu W., Xu X.M. (2017). Optogenetics and its application in neural degeneration and regeneration. Neural. Regen Res..

[247] Lin J.Y., Knutsen P.M., Muller A., Kleinfeld D., Tsien R.Y. (2013). ReaChR: A red-shifted variant of channelrhodopsin enables deep transcranial optogenetic excitation. Nat. Neurosci..

[248] Klapoetke N.C., Murata Y., Kim S.S., Pulver S.R., Birdsey-Benson A., Cho Y.K., Morimoto T.K., Chuong A.S., Carpenter E.J., Tian Z. (2014). Independent optical excitation of distinct neural populations. Nat. Methods.

[249] Kim C.K., Yang S.J., Pichamoorthy N., Young N.P., Kauvar I., Jennings J.H., Lerner T.N., Berndt A., Lee S.Y., Ramakrishnan C. (2016). Simultaneous fast measurement of circuit dynamics at multiple sites across the mammalian brain. Nat. Methods.

[250] Park D.W., Schendel A.A., Mikael S., Brodnick S.K., Richner T.J., Ness J.P., Hayat M.R., Atry F., Frye S.T., Pashaie R. (2014). Graphene-based carbon-layered electrode array technology for neural imaging and optogenetic applications. Nat. Commun..

[251] Renault R., Sukenik N., Descroix S., Malaquin L., Viovy J.L., Peyrin J.M., Bottani S., Monceau P., Moses E., Vignes M. (2015). Combining microfluidics, optogenetics and calcium imaging to study neuronal communication in vitro. PLoS ONE.

[252] Zhang F., Gradinaru V., Adamantidis A.R., Durand R., Airan R.D., de Lecea L., Deisseroth K. (2010). Optogenetic interrogation of neural circuits: Technology for probing mammalian brain structures. Nat. Protoc..

[253] Huber D., Petreanu L., Ghitani N., Ranade S., Hromádka T., Mainen Z., Svoboda K. (2008). Sparse optical microstimulation in barrel cortex drives learned behaviour in freely moving mice. Nature.

[254] Grosenick L., Marshel J.H., Deisseroth K. (2015). Closed-loop and activity-guided optogenetic control. Neuron.

[255] Gagnon-Turcotte G., LeChasseur Y., Bories C., Messaddeq Y., De Koninck Y., Gosselin B. (2017). A Wireless Headstage for Combined Optogenetics and Multichannel Electrophysiological Recording. IEEE Trans. Biomed. Circuits Syst.

[256] Pansare V., Hejazi S., Faenza W., Prud’homme R.K. (2012). Review of Long-Wavelength Optical and NIR Imaging Materials: Contrast Agents, Fluorophores and Multifunctional Nano Carriers. Chem. Mater..

[257] Yue L., Weiland J.D., Roska B., Humayun M.S. (2016). Retinal stimulation strategies to restore vision: Fundamentals and systems. Prog. Retin. Eye Res..

[258] Bareket L., Barriga-Rivera A., Zapf M.P., Lovell N.H., Suaning G.J. (2017). Progress in artificial vision through suprachoroidal retinal implants. J. Neural Eng..

[259] Rizzo S., Cinelli L., Finocchio L., Tartaro R., Santoro F., Gregori N.Z. (2019). Assessment of Postoperative Morphologic Retinal Changes by Optical Coherence Tomography in Recipients of an Electronic Retinal Prosthesis Implant. JAMA Ophthalmol..

[260] Hahn P., Fine H.F. (2018). Practical Concepts With the Argus II Retinal Prosthesis. Ophthalmic Surg. Lasers Imaging Retina.

[261] Rachitskaya A., Lane L., Ehlers J., DeBenedictis M., Yuan A. (2019). Argus II Retinal Prosthesis Implantation Using Three-Dimensional Visualization System. Retina.

[262] Stingl K., Bartz-Schmidt K.U., Besch D., Chee C.K., Cottriall C.L., Gekeler F., Groppe M., Jackson T.L., MacLaren R.E., Koitschev A. (2015). Subretinal Visual Implant Alpha IMS--Clinical trial interim report. Vision Res..

[263] Kuehlewein L., Troelenberg N., Stingl K., Schleehauf S., Kusnyerik A., Jackson T.L., MacLaren R.E., Chee C., Roider J., Wilhelm B. (2019). Changes in microchip position after implantation of a subretinal vision prosthesis in humans. Acta Ophthalmol..

[264] Rachitskaya A., Yuan A., Davidson S., Streicher M., DeBenedictis M., Rosenfeldt A.B., Alberts J. (2020). Computer-Assisted Immersive Visual Rehabilitation in Argus II Retinal Prosthesis Recipients. Ophthalmol. Retina.

[265] Winter J.O., Cogan S.F., Rizzo J.F. (2007). Retinal prostheses: Current challenges and future outlook. J. Biomater. Sci. Polym. Ed..

[266] Dagnelie G., Christopher P., Arditi A., da Cruz L., Duncan J.L., Ho A.C., Olmos de Koo L.C., Sahel J.A., Stanga P.E., Thumann G. (2017). Performance of real-world functional vision tasks by blind subjects improves after implantation with the Argus® II retinal prosthesis system. Clin. Exp. Ophthalmol..

[267] Kitiratschky V.B., Stingl K., Wilhelm B., Peters T., Besch D., Sachs H., Gekeler F., Bartz-Schmidt K.U., Zrenner E. (2015). Safety evaluation of “retina implant alpha IMS”—A prospective clinical trial. Graefes Arch. Clin. Exp. Ophthalmol..

[268] Kienzler M.A., Isacoff E.Y. (2017). Precise modulation of neuronal activity with synthetic photoswitchable ligands. Curr. Opin. Neurobiol..

[269] Tochitsky I., Kienzler M.A., Isacoff E., Kramer R.H. (2018). Restoring Vision to the Blind with Chemical Photoswitches. Chem. Rev..

[270] Polosukhina A., Litt J., Tochitsky I., Nemargut J., Sychev Y., De Kouchkovsky I., Huang T., Borges K., Trauner D., Van Gelder R.N. (2012). Photochemical restoration of visual responses in blind mice. Neuron.

[271] Tochitsky I., Polosukhina A., Degtyar V.E., Gallerani N., Smith C.M., Friedman A., Van Gelder R.N., Trauner D., Kaufer D., Kramer R.H. (2014). Restoring visual function to blind mice with a photoswitch that exploits electrophysiological remodeling of retinal ganglion cells. Neuron.

[272] Lerch M.M., Hansen M.J., van Dam G.M., Szymanski W., Feringa B.L. (2016). Emerging Targets in Photopharmacology. Angew. Chem. Int. Ed. Engl..

[273] Shim G., Ko S., Kim D., Le Q.V., Park G.T., Lee J., Kwon T., Choi H.G., Kim Y.B., Oh Y.K. (2017). Light-switchable systems for remotely controlled drug delivery. J. Control. Release.

[274] Ma Y., Bao J., Zhang Y., Li Z., Zhou X., Wan C., Huang L., Zhao Y., Han G., Xue T. (2019). Mammalian Near-Infrared Image Vision through Injectable and Self-Powered Retinal Nanoantennae. Cell.

[275] Hüll K., Benster T., Manookin M.B., Trauner D., Van Gelder R.N., Laprell L. (2019). Photopharmacologic Vision Restoration Reduces Pathological Rhythmic Field Potentials in Blind Mouse Retina. Sci. Rep..

